# Two Decades of Human–Elephant Conflict in Jharkhand: Spatial and Ecological Drivers of Human Fatalities

**DOI:** 10.1002/ece3.72679

**Published:** 2025-12-18

**Authors:** Kalpana Roy, Ramesh Kumar Pandey, Athira N. Ganesan, Ananya Dutta, Dheeraj Mittal, Parag Nigam, Anukul Nath, Bilal Habib

**Affiliations:** ^1^ Wildlife Institute of India Dehradun India; ^2^ Ministry of Environment Forest and Climate Change (MoEFCC) New Delhi India; ^3^ Academy of Scientific and Innovative Research (AcSIR) Ghaziabad India

**Keywords:** conflict, elephant, human fatalities, monsoon, Ranchi

## Abstract

Human–Elephant Conflict (HEC) is a major issue in South Asia, especially in India, where rapid development and increased human–elephant overlap intensify the problem. We analyzed human fatality and injury patterns over 23 years (2000–2023) in Jharkhand, identified influencing factors, and prioritized villages for targeted mitigation. We collected data from 22 Forest Divisions, comprising 1740 incidents, including 1340 fatalities and 400 injuries, predominantly in Ranchi, Khunti, and East Singhbhum. The rise in conflict incidents during the monsoon season could be associated with poor visibility. Spatial analysis revealed that conflict hotspots are concentrated near protected areas and fragmented forest habitats. High‐conflict villages exhibit greater cropland and built‐up areas, while medium‐ and low‐conflict villages show variability in land use patterns. Landscape fragmentation metrics, such as forest patch density, are significantly associated with conflict intensity, emphasizing the role of habitat degradation and fragmentation. Proximity to forests, water sources, roads, and elephant reserves strongly influences conflict risks. While urbanization disrupts elephant corridors, forest‐edge areas near agricultural zones are critical conflict zones. Therefore, mitigation strategies must prioritize high‐conflict villages, including villages Ramua, Chatambari, Gerapokhar, Gagi, Bhatin, and Koderma, through targeted interventions. Our study highlights the necessity of integrating ecological and socio‐economic dimensions into conflict mitigation, emphasizing habitat restoration, land use, and targeted infrastructure planning, and community engagement. By implementing such measures, it is possible to reduce the underlying drivers of HEC, thereby safeguarding human lives while ensuring elephant conservation and contributing to broader biodiversity and ecosystem health goals.

## Graphical abstract

Conflict hotspot (human fatalities and injuries) from 2000 to 2023 in Jharkhand.

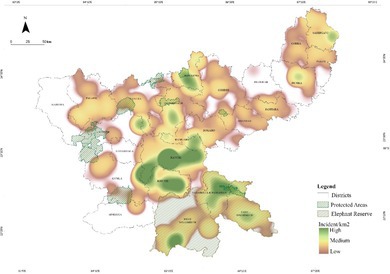

## 1 Introduction

Human‐Elephant Conflict (HEC) has become one of the most significant issues in Asia, primarily in India, Sri Lanka, and Nepal, where expanding human populations and infrastructure developments have invaded natural habitats (Shaffer et al. 2019). HEC refers to the negative interactions between humans and elephants, typically resulting in damage to crops, property, and infrastructure, as well as injuries and fatalities for both humans and elephants (Sukumar 2006). In South Asia, the distribution of HEC is influenced by the large elephant populations and increased human encroachment into forested regions (Choudhury 2004; Fernando et al. 2005). This overlap has resulted in a significant rise in conflicts in areas where elephants traditionally venture. For example, in Sri Lanka, HEC is most prevalent in the dry zones, where elephants' home ranges overlap with intensive paddy cultivation (Fernando et al. 2023). Similarly, in Nepal, elephants raid crops near buffer zones of national parks, leading to rising death tolls and monetary losses (Pant et al. 2016).

India holds around 60% of the global Asian elephant population (Baskaran et al. 2011). States such as Assam, Odisha, Karnataka, and Jharkhand experience high rates of conflict due to habitat fragmentation and the expansion of human settlement (Chartier et al. 2011). According to the 2017 elephant census, Jharkhand had an estimated 679 Asian elephants (MoEFCC 2019). In Jharkhand, HEC has sharply increased in the past two decades due to deforestation, mining, industrialization, and rapid urbanization (Dash et al. 2024). Jharkhand served as a natural habitat and migratory corridor for elephants between the forests of West Bengal, Odisha, and Chhattisgarh (Kanga et al. 2017). However, extensive coal mining operations, particularly in districts such as Dhanbad, Hazaribagh, and West Singhbhum, have led to significant habitat fragmentation (Pande et al. 2015). The degradation of important elephant corridors, such as the Saranda Forest, which acts as a critical migration route for elephant movement in Odisha and Jharkhand, has further disrupted elephant migration routes (Rangarajan et al. 2010). As elephants are pushed into human settlements, incidents of crop‐raiding, property damage, and human fatalities have surged, creating a significant burden for local communities that rely on agriculture and forest resources for their livelihoods (Dash et al. 2024). The extensive deforestation and fragmentation of natural habitats in Jharkhand, driven by mining activities, have forced elephants into closer proximity to human settlements. Between 2001 and 2020, the state lost nearly 8000 ha of forest cover, with districts such as West Singhbhum and Giridih experiencing the highest levels of deforestation (Global Forest Watch 2023). As a result, traditional migratory routes for elephants, which once connected the forests of the Chotanagpur plateau to neighboring regions, have been disrupted. The reduction in forest cover has also led to the degradation of key elephant corridors, such as those linking Jharkhand with Odisha's Simlipal National Park (Debata et al. 2013). This has not only increased the frequency of HEC incidents but also threatens the long‐term survival of elephants by limiting their access to critical habitats and reducing genetic diversity within populations (Baskaran 1998).

Communities living in and around these forested areas are particularly vulnerable to the impacts of HEC. Many of these communities, especially indigenous tribes, rely on forest resources for food, fuel, and medicine (Rao and Vishwanath 2007). The loss of crops and property due to elephant raids has severe economic consequences for these households, many of which have limited alternative sources of income (Weinmann 2018). Despite government efforts to provide compensation for losses incurred through HEC, many community members report that these payments are insufficient and delayed (Guru and Das 2021). As a result, frustration and resentment toward elephants have grown, complicating efforts to promote human–wildlife coexistence. Mining and industrial development in Jharkhand have not only fragmented elephant habitats but have also increased human presence in these areas, further escalating the likelihood of conflict (Sonter et al. 2018). Mines tend to create large labour demand, and as a consequence, the presence of mines tends to trigger labour in migration into mining regions. These areas often become hotspots for HEC as elephants, in search of food and water, raid crops and homes (Saini 2018). The disruption of elephant corridors, such as the Saranda Forest corridor, exacerbates these conflicts by limiting the elephants' ability to move between forested areas (Menon 2017).

The degradation of elephant habitats in Jharkhand has far‐reaching consequences for both elephants and humans. Elephants that are unable to access traditional migratory routes are forced into fragmented habitats, increasing the likelihood of inbreeding and reducing genetic diversity within populations (De et al. 2021; Nad et al. 2022; Pandey, Lakshminarayanan, et al. 2024). This, in turn, can negatively impact the health and survival of elephant populations in the long term (Baskaran et al. 2013). For local communities, the economic and social impacts of HEC are severe. Crop losses, property damage, and human fatalities have become common occurrences, particularly in districts such as West Singhbhum, Giridih, and Hazaribagh (Dash et al. 2024). Human injuries or deaths caused by elephants are rare but often result from accidental encounters, such as crossing paths near water bodies, being too close to distressed or aggressive elephants, or during conflicts over crop protection (Lingaraju and Venkataramana 2014). These incidents, though infrequent compared to other causes of mortality like malaria or road accidents, generate fear in rural communities and hinder conservation efforts (Lingaraju and Venkataramana 2014). Factors contributing to such conflicts include blocked traditional routes, harassment, and settlements encroaching on elephant habitats. Studies from India and Sri Lanka comprehensively document human fatalities caused by HEC and emphasize the urgent need for expanded, multi‐faceted research to understand and mitigate these conflicts more effectively (Fernando et al. 2005; Gubbi 2012; Shaffer et al. 2019; Perera et al. 2022; Pandey, Yadav, et al. 2024). However, studies on the factors governing human fatalities due to HEC are limited. Therefore, in the present study, we investigate the spatial, temporal, and demographic patterns of human fatalities caused by HEC in Jharkhand. Here, we identify the key influencing factors and assess villages in order to inform the prioritization of targeted mitigation measures by relevant authorities.

### 1.1 Study Area

The eastern Indian state of Jharkhand encompasses 79,716 km^2^, rich in natural resources, with densely covered forest areas that have emerged as key habitats for Asian elephants. The topography here varies and ranges from hills and plateaus to valleys, bringing together a diverse landscape on the scale of balances between forest regions and agricultural and mining areas. The geographical region of Jharkhand is marked by forest cover, comprising circa 29% of its total geographical area (FSI 2021). Most such areas consist of tropical dry deciduous forests, tropical moist deciduous forests, and several stretches of sal forests ( *Shorea robusta* ). These forests are therefore elemental to the very survival of elephants in the region because they provide food, shelter, and migration routes—all of which are fundamental to their movement at the change of seasons.

The state experiences a subtropical climate with a monsoon period from June to September. The average annual precipitation is approximately 1398 mm, predominantly received during the monsoon season, while the temperature ranges from 5°C in winter to 45°C in summer (Kumar et al. 2013). The state's population, as per the 2011 Census, is around 32.96 million, with a sex ratio of 947 females per 1000 males (Census of India 2011). The literacy rate is 67.63%, with significant rural–urban disparities. Educational attainment among tribal communities remains a key area of concern due to systemic challenges (Kumar 2008). Jharkhand has a substantial tribal population, constituting about 26.21% of its total population. These communities often rely on subsistence agriculture and forest resources, with a significant proportion holding marginal or small landholdings. Socio‐economic studies reveal that annual incomes for many tribal households are below ₹30,000, and unemployment rates remain high in these communities (Islam et al. 2014). Furthermore, the state has seen varying impacts of socio‐economic development, influenced by factors such as education, health infrastructure, and access to resources (Prakash 1998).

Saranda Forest, Palamu Tiger Reserve, and Dalma Wildlife Sanctuary are important elephant habitats in the state. Besides being a habitat for elephants, these areas also act as corridors for the migration of elephants from one state to the neighboring states like West Bengal, Odisha, and Chhattisgarh (Sukumar 2006). There is a seasonal migration of elephants at pre‐monsoon and post‐monsoon times in search of water and food by elephants (Anoop et al. 2023). These movements often bring elephants into closer proximity with human settlements, increasing the likelihood of conflict (Choudhury 2004). The presence of bamboo, grass, and fruiting trees during these seasons in the forests of Jharkhand draws the elephants to those areas, but habitat fragmentation increases the chances of conflict with the local communities (Sukumar 2006).

Industrialization in the form of mining and infrastructure development has considerably altered the ancient migratory routes of the elephants over the last few decades. Jharkhand is the biggest coal and iron ore‐producing state in the country, with numerous mines located either inside or in proximity to the large elephant habitats (Saini et al. 2016).

## 2 Methodology

### 2.1 Collection of HEC Occurrences

**FIGURE 1:**
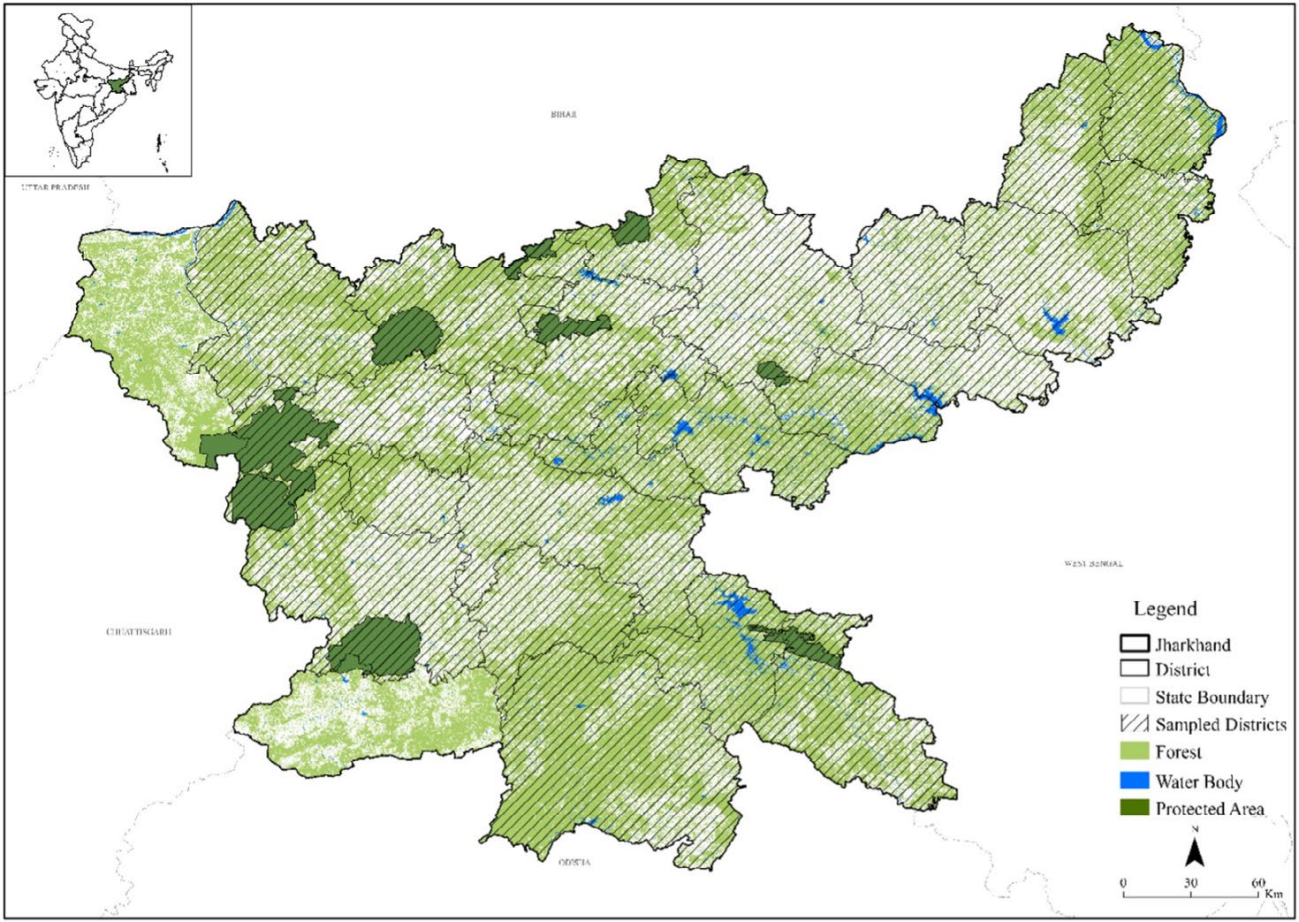
Map of Jharkhand, India, showing protected areas and the locations of human fatalities and injuries from human–elephant conflict during 2000–2023.

Data on HEC occurrences was gathered from 22 Divisional Forest Offices in Jharkhand (Figure 1), covering the period from 2000 to 2023. The available information from the department included the name of the division, village, the date of the incidents, human fatalities, and injuries (including gender). Incident locations, provided as geographic coordinates by the Forest Department, were used to develop a spatial distribution of conflict incidents. Land use land cover, spatial pattern of HEC, and forest fragmentation.

The dataset on HEC comprised 1740 cases of human deaths and injuries caused by wild elephants over a span of 23 years. We divided the data into 5‐year intervals grouped as 2000–2005, 2006–2010, 2011–2015, 2016–2020, 2021–2023. The Land Use Land Cover (LULC) map of Jharkhand state was created using Landsat 5 TM and Landsat 8 OLI imagery for the respective years. The Human‐Elephant Conflict (HEC) incident data were categorized by death and injury, gender, year, season, and division. To understand the spatial distribution of conflict, we mapped the conflict hotspot using a kernel density estimator, using ArcGIS Pro with an output cell size of 200 m to account for geolocation accuracy. In addition, landscape fragmentation was analyzed using FRAGSTATS (v4.2) to calculate key landscape metrics. The input data, derived from LULC maps, were reclassified into forest and non‐forest classes using the Spatial Analyst tool. To avoid redundancy and enhance interpretability, metrics that only effectively capture important landscape features were selected. Class‐level metrics for forest cover, such as Patch Density (PD), Edge Density (ED), and Largest Patch Index (LPI), were calculated. A 7 km moving window analysis, based on the average movement of elephants (Hassan et al. 2023), was used to generate a continuous surface, ensuring ecologically relevant outcomes. To analyze the factors influencing HEC events, spatial data were utilized for variables including distances to forests, croplands, built‐up areas, roads, waterways, protected areas, elephant reserves, and mines.

The shortest distances between conflict points and these features were calculated using the “Generate Near Table” tool in ArcGIS Pro. These distance values, along with the above‐mentioned fragmentation metrics, were subsequently employed as predictor variables and human fatalities and injuries as response variables. We used a candidate regression model using Generalized Linear Models (GLMs) along with the “MuMIn” package in *R* for model selection. The models were built based on a priori hypotheses, ensuring the inclusion of variables with theoretical relevance to HEC (Table 1). Prior to model building, all the variables were z‐transformed and checked for multicollinearity (Figure S1). Models were ranked using the Akaike Information Criterion (AIC). Cross‐validation techniques evaluated model robustness, and the optimal model was selected by averaging candidate models with ΔAIC ≤ 2. Each HEC event involving human fatalities was coded as 1, while pseudo‐points generated locations that represent areas without recorded conflict were randomly assigned a value of 0. A total of 1740 pseudo‐absence points were generated using ArcGIS Pro, at least 1 km away from actual conflict points, to ensure spatial relevance while avoiding overlap.

**TABLE 1:** A priori hypotheses for all environmental variables correlating human deaths and injuries by elephants.

Feature	Variable	Description and source	A priori hypothesis
Landcover	Distance from built‐up (db)	Distance from landcover features are extracted using Near Table tool (ArcPro 3.0.0).	Proximity to built‐up areas increases HEC due to habitat loss and higher human activity (Sukumar 1990; Hoare 2015).
	Distance from cropland (dc)		Proximity to croplands increases HEC due to crop‐raiding by elephants (Sukumar 2003; Gubbi 2012).
	Distance from forest (df)		Proximity to forests increases HEC as fragmented forests bring elephants closer to human settlements (Goswami et al. 2015; Mumby and Plotnik 2018).
	Distance from waterbodies (dw)		Proximity to waterbodies increases HEC as elephants seek water, especially in dry seasons (Sukumar 2003; Perera 2009).
	Distance from mines and quarries (dmn)	Distance from mines and quarries digitized using Google Earth Pro and Near Table tool (ArcPro 3.0.0).	Proximity to mines increases HEC due to habitat fragmentation and human disturbances.
Anthropogenic	Distance from road (dr)	Distance from roads using OpenStreetMap.org data and Near Table tool (ArcPro 3.0.0).	Proximity to roads increases HEC due to habitat fragmentation and human‐elephant interactions (Forman and Alexander 1998; Gubbi 2012).
	Distance from protected areas (dpa)	Distance from protected areas using shapefiles provided by the Elephant Cell, WII, and Near Table tool (ArcPro 3.0.0).	Proximity to protected areas increases HEC due to elephants venturing out for resources (Chartier et al. 2011).
	Distance from elephant reserves (dr)	Distance from elephant reserves using Near Table tool (ArcPro 3.0.0).	Proximity to reserves increases HEC as elephants move between reserves and human settlements (Sukumar 2003; Leimgruber et al. 2003).
	Edge density (ed)	Edge density (ED) and patch density (PD) calculated using FRAGSTATS 4.2.	Higher edge density increases HEC due to greater human‐elephant interfaces (McGarigal and Marks 1995; Karanth et al. 2012)
Landscape Metrics	Patch density (pd)	Edge density (ED) and patch density (PD) calculated using FRAGSTATS 4.2.	Higher patch density increases HEC due to habitat fragmentation and disrupted elephant movement patterns (McGarigal and Marks 1995; Cushman et al. 2010).

### 2.2 Village Level Analysis for Highlighting Prioritization Villages for Mitigation

To assess the intensity of HEC, we analyzed the frequency of conflict incidents reported across all villages within the forest divisions. Based on the occurrence rates, villages were categorized into three levels of conflict intensity: high (> 20 conflict incidents), medium (11–20), and low (1–10). The percentage of each ecological and anthropogenic variable—water, built‐up, road density, forest, crop, and mines percentage was calculated for each village. We performed a nonparametric Kruskal‐Wallis test to test whether these predictor variables vary significantly across incident groups (incident, non‐incident, high incident, medium incident, low incident). The village boundaries were obtained from ArcGIS Online, shapefile: Indian Administrative Layer 2024.

## 3 Results

### 3.1 Temporal and Seasonal Patterns

**FIGURE 2:**
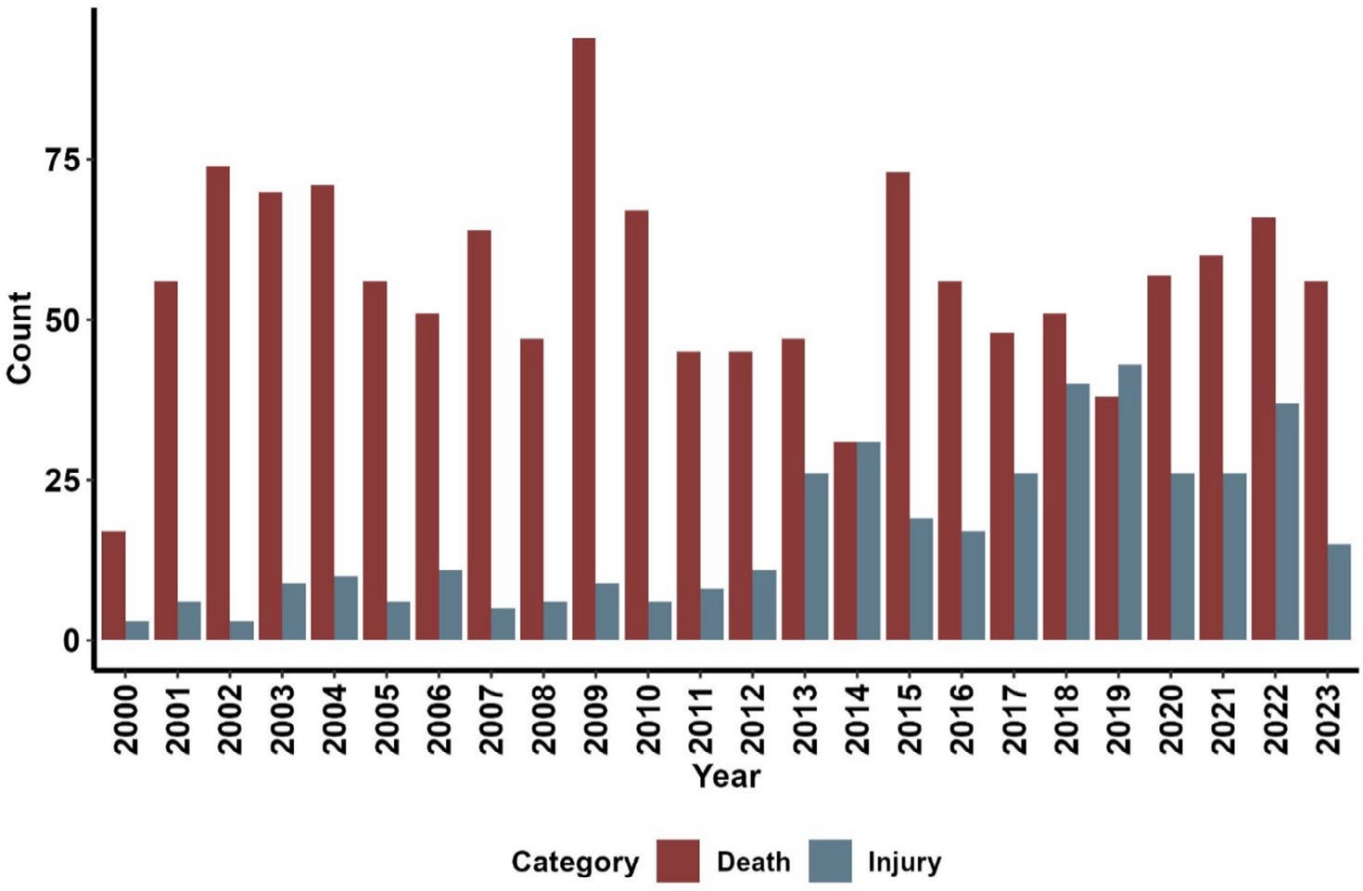
Trends in human fatalities and injuries caused by human–elephant conflict in Jharkhand, India (2000–2023).

**FIGURE 3:**
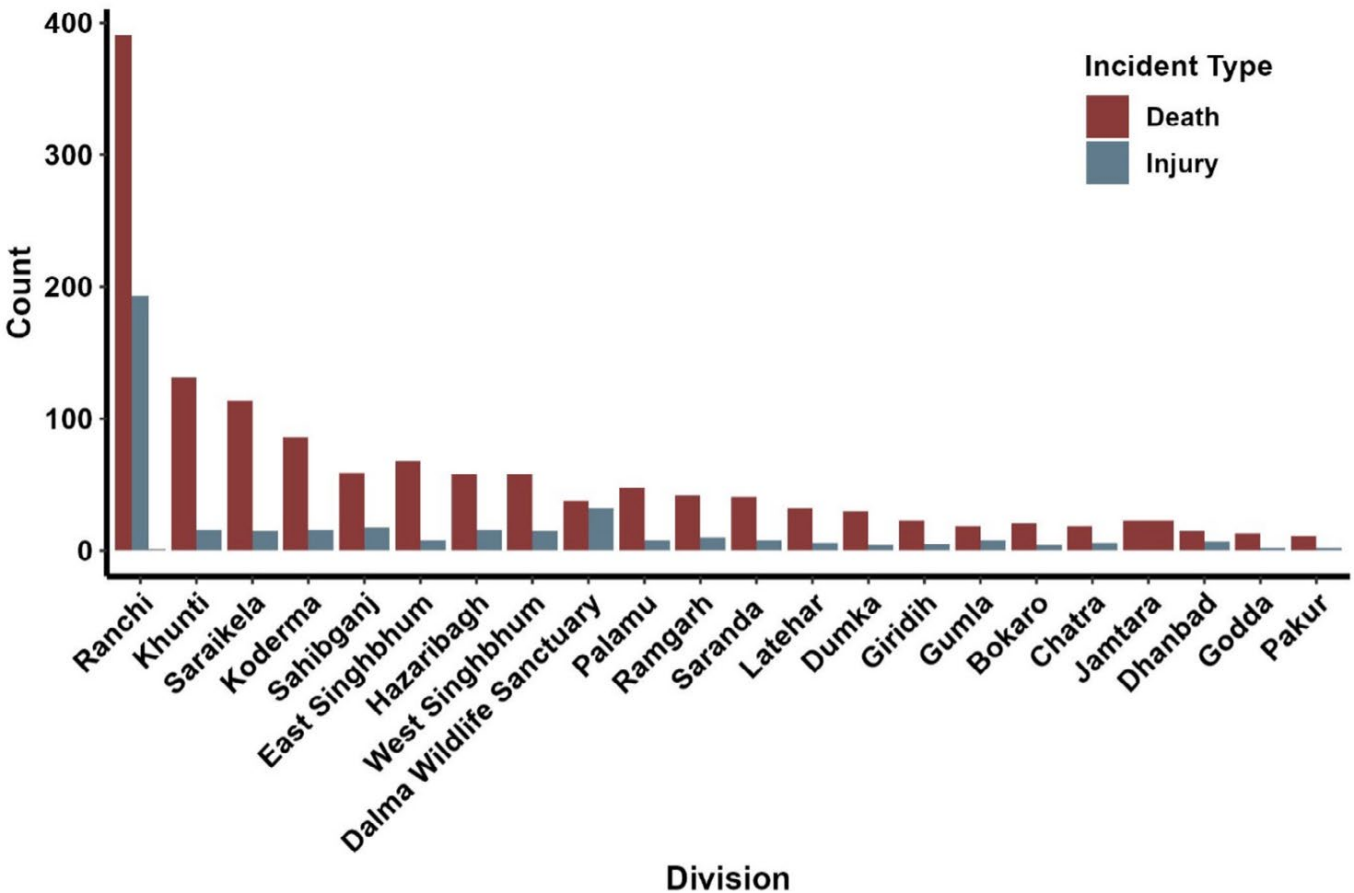
Division‐wise distribution of human‐elephant incidents in Jharkhand, India from 2000 to 2023.

**FIGURE 4:**
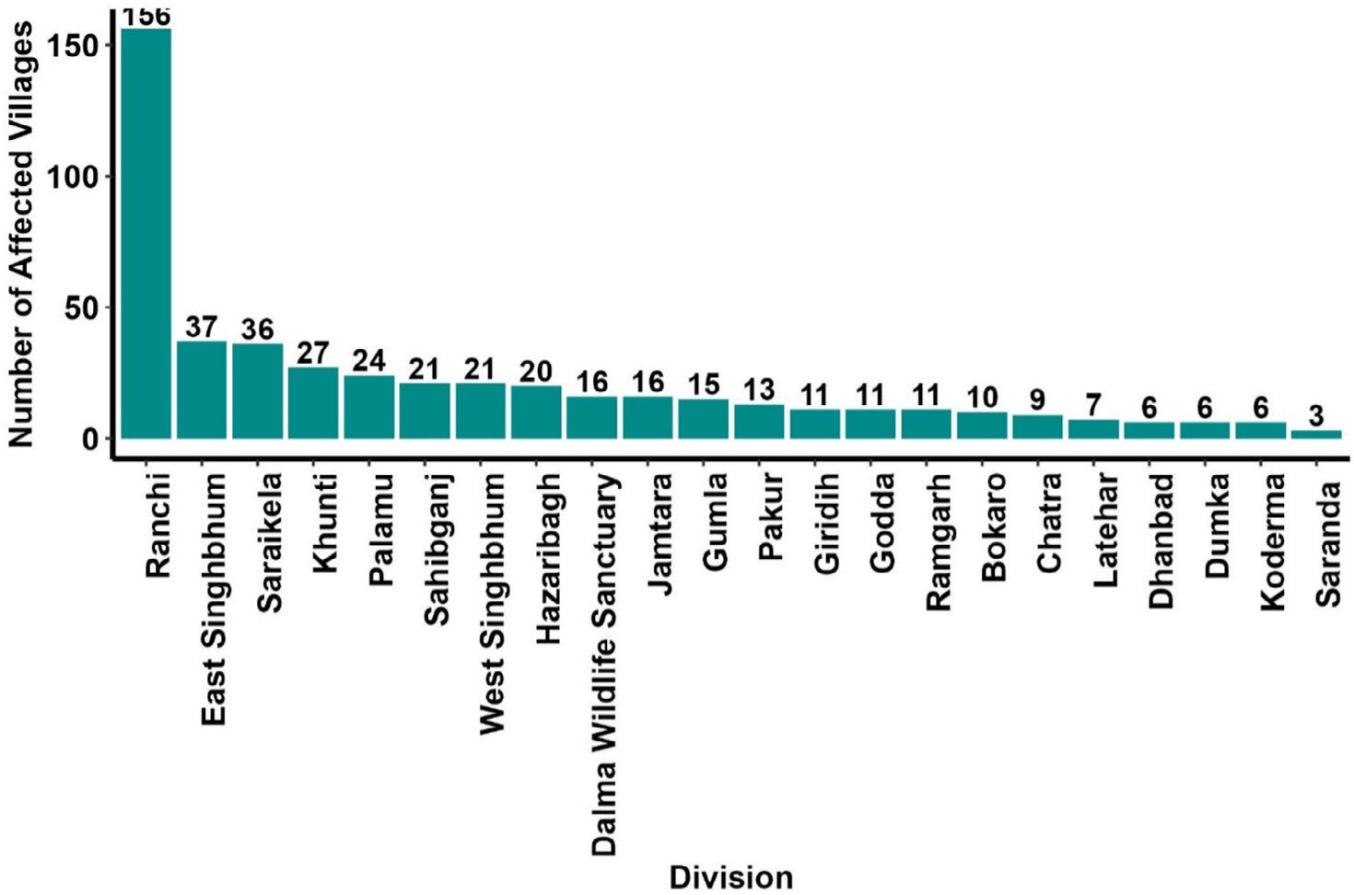
Number of villages affected by human‐elephant conflict per division in Jharkhand, India (2000–2023).

**FIGURE 5:**
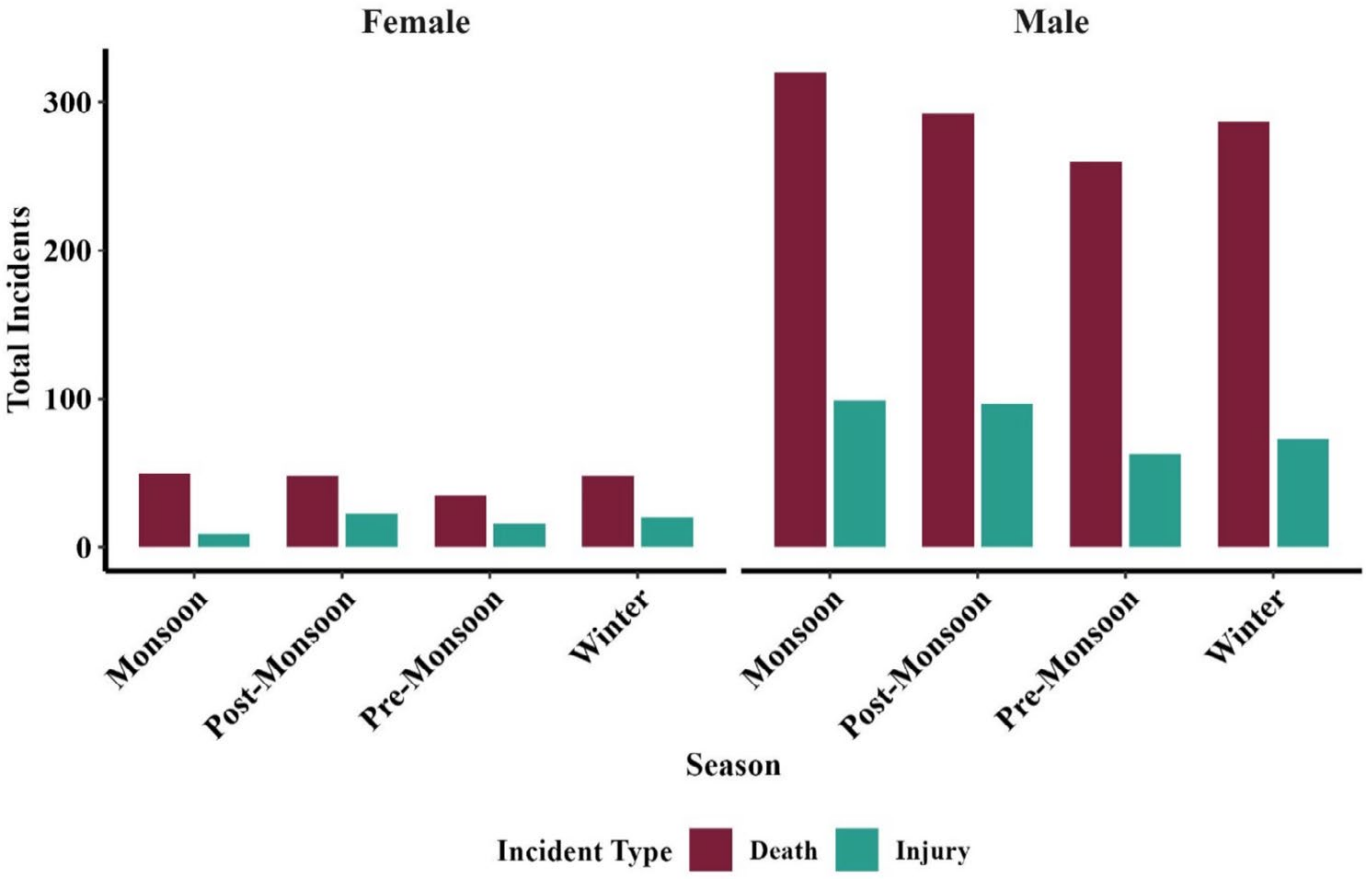
Seasonal variation in human fatalities/injuries (male and female) in the state of Jharkhand, India, during 2000–2023.

Between 2000 and 2023, Jharkhand recorded 1740 incidents, resulting in 1340 human fatalities and 400 injuries. Fatalities peaked in 2014, but no significant long‐term trend was observed (𝜏 = −0.08, *p* = 0.60). In contrast, injuries showed a statistically significant increasing trend (𝜏 = 0.64, *p* < 0.001) (Figure 2). Ranchi was the most affected division (391 deaths, 194 injuries), followed by Khunti (131 deaths,16 injuries), East Singhbhum (68 deaths, 8 injuries), Hazaribagh (58 deaths, 16 injuries) and Palamu (48 deaths, 8 injuries) (Figure 3). Kernel density estimator using spatial location also revealed conflict hotspots in Ranchi, Khunti, and East Singhbhum, with expanding conflict areas including protected regions like Hazaribagh, Palamu, and Dalma Wildlife Sanctuary (Figure 4). The fatalities and injuries reported significantly higher in males compared to females (*χ* ^2^ = 886.53, df = 1, *p* < 0.0001; Figure 5). Casualties were also higher in the monsoon season.

**FIGURE 6:**
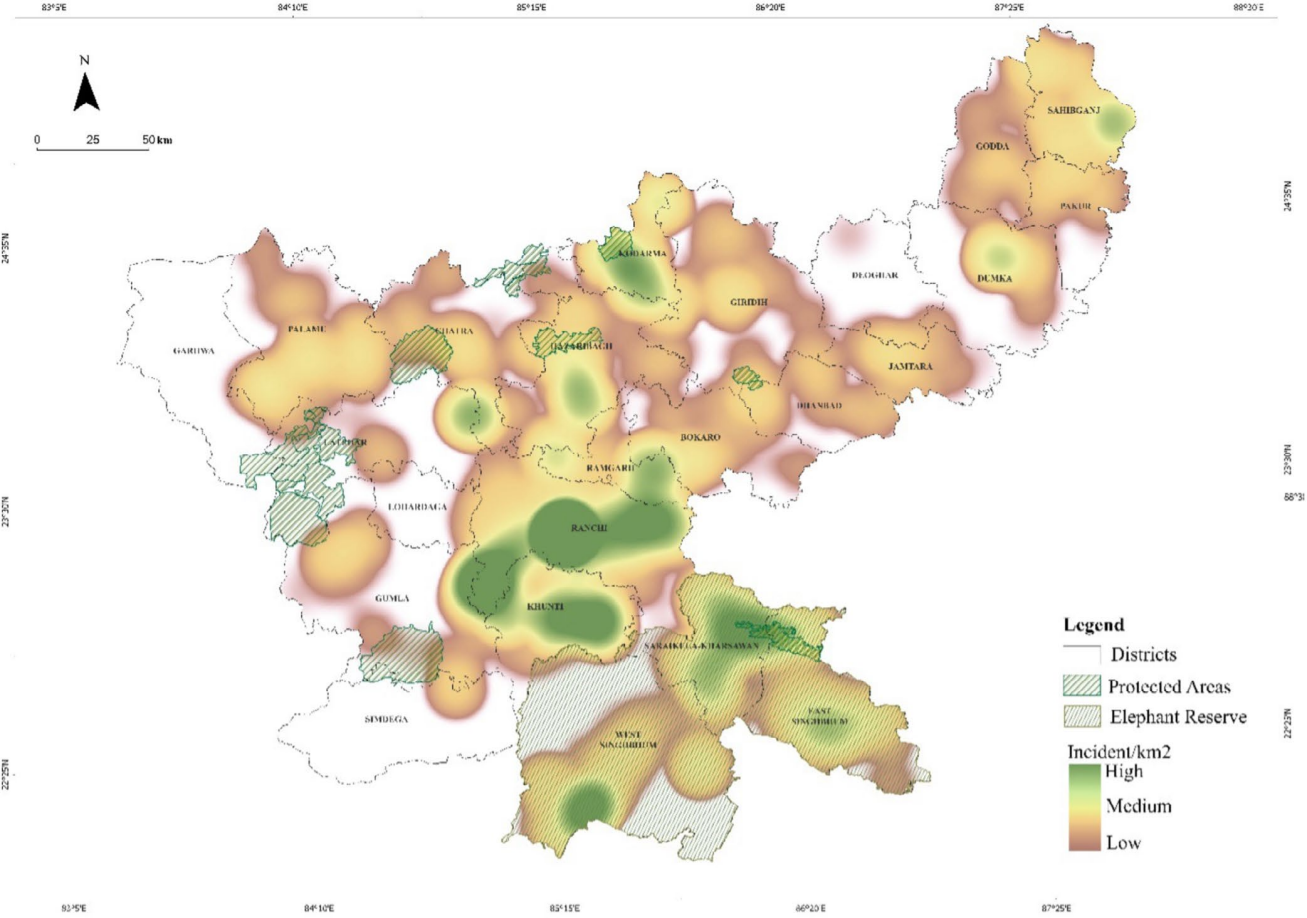
Hotspots of human fatalities and injuries in the State of Jharkhand, India from 2000 to 2023. The map was created using the kernel density tool in ArcGIS Pro version 3.0.0.

At present, 480 villages of Jharkhand were affected by HEC. Ranchi recorded the maximum number of affected villages (156), followed by East Singhbhum (37) and Saraikela (36) (Figure 6). Other divisions, including Khunti, Palamu, Sahibganj, West Singhbhum, Hazaribagh, and Dalma Wildlife Sanctuary, also reported frequent incidents. Other Forest Divisions like Gumla, Pakur, and Giridih experienced comparatively fewer incidents.

### 3.2 Ecological and Anthropogenic Drivers of HEC

**FIGURE 7:**
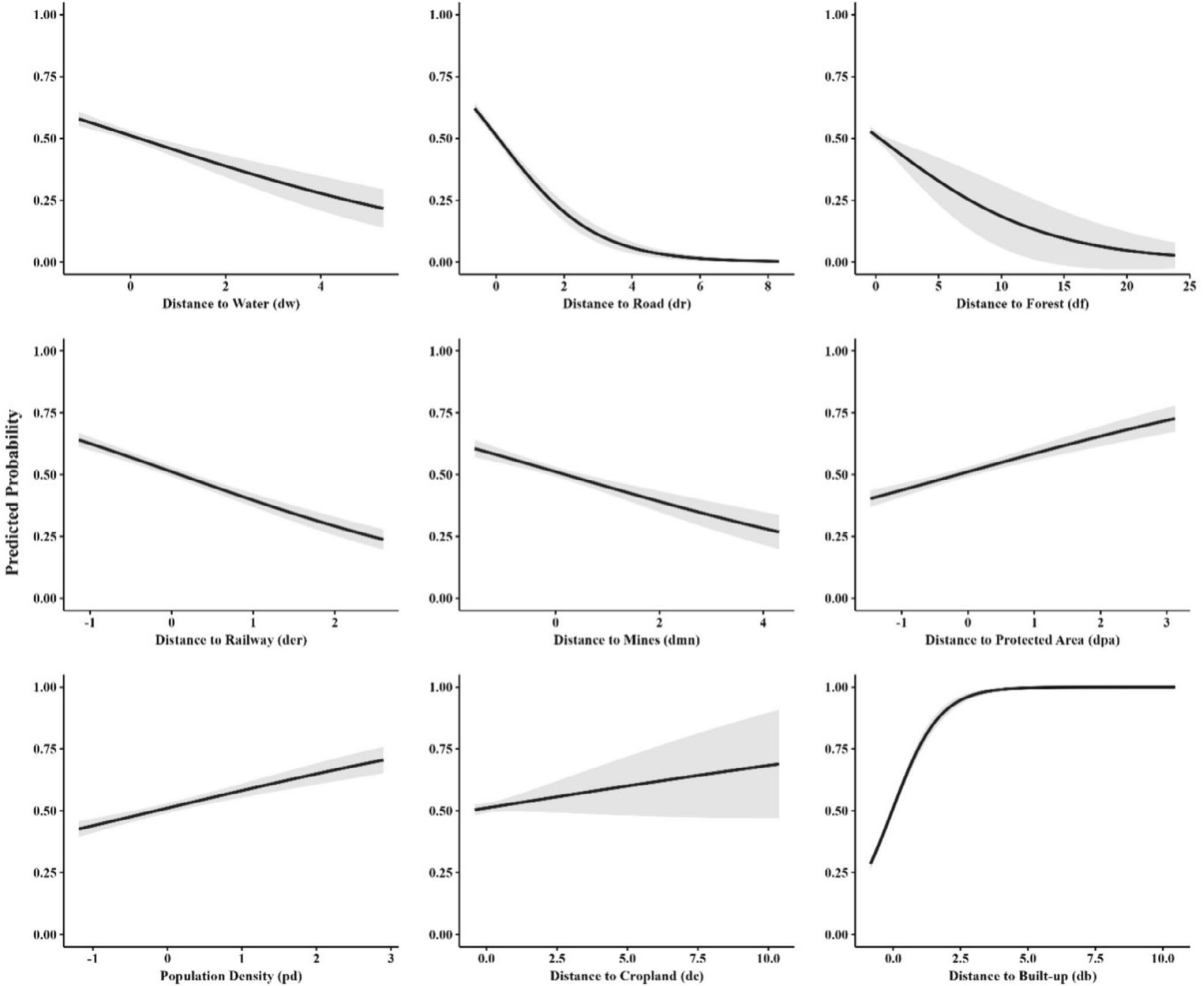
Response curves depicting the probability of human fatalities and injuries based on proximity to features, influencing the likelihood of HEC in the state of Jharkhand, India from 2000 to 2023.

We found that proximity to both natural features (forests, water bodies, and elephant reserves) and human‐modified landscapes (roads, crop fields, and built‐up areas) was a critical factor influencing human fatalities and injuries (Tables 2 and 3, Figure 7). As expected, conflict incidences were higher closer to water bodies (*β* = −0.007, *p* < 0.001), roads (*β* = −0.906, *p* < 0.001), and elephant reserves (*β* = −0.878, *p* < 0.001), highlighting the spatial overlap of human and elephant activity in these areas. Areas closer to forests also showed increased risk (*β* = −0.003, *p* < 0.001), emphasizing the importance of forest edges as conflict zones. The analysis revealed high conflict probability near mines (*β* = −0.101, *p* < 0.001). However, conflict incidence decreased with an increase in distance from built‐up areas (*β* = 0.143, *p* < 0.001) and protected areas (*β* = 0.011, *p* < 0.001). Landscape configuration also influenced conflict patterns, with higher fragmented forest patch density (*β* = 0.220, *p* < 0.001) associated with increased conflict risk.

**TABLE 2:** Summary statistics loglikelihood (LogL), degrees of freedom (df), Akaike Information Criteria (AICc), relative support for hypothesis (ΔAICc), Akaike weights (Wi) of candidate regression model explaining HEC in Jharkhand.

Model description	LogL	df	AICc	ΔAICc	Wi
dw + dr + df + der + dmn + dpa + pd. + dc + db	−1925.23	10	3870.52	0	0.51
dw + dr + df + der + dmn + dpa + pd. + db	−1926.29	9	3870.629	0.109	0.49
dr + df + der + dmn + dpa + pd. + db	−1943.48	8	3902.992	32.472	0.00
dw + dr + df + der + dpa + pd. + db	−1945.61	8	3907.259	36.739	0.00
dw + dr + df + der + dmn + pd. + db	−1952.93	8	3921.909	51.389	0.00
dr + df + dmn + db	−2071.05	5	4152.118	281.598	0.00
dw + dpa + pd. + db	−2151.75	5	4313.521	443.001	0.00
der + db	−2183.69	3	4373.378	502.858	0.00
dr + df + der + dmn + dpa + pd	−2190.17	7	4394.366	523.846	0.00
dr + df + der + dmn + dpa	−2203.85	6	4419.717	549.197	0.00
dw + dr + df + dpa + pd	−2266.12	6	4544.254	673.733	0.00
dr + dpa + dc	−2276.13	4	4560.272	689.752	0.00
dw + der + dmn + pd	−2277.51	5	4565.034	694.513	0.00
dr + dpa	−2289.96	3	4585.918	715.398	0.00
dw + dr + pd	−2295	4	4598.014	727.494	0.00
dw + df + der	−2324.18	4	4656.379	785.859	0.00
Intercept only	−2412.15	1	4826.306	955.785	0.00

**TABLE 3:** Summary statistics of model with lowest AICc (dw + dr + df + der + dmn + dpa + pd. + dc + db).

Predictor variable	Beta coefficient (*β*)	Std. error	*z*	*p*
(Intercept)	0.043	0.040	1.071	0.28
Distance to waterbodies (dw)	−0.250	0.042	−5.835	0.001
Distance to roads (dr)	−0.708	0.054	−12.992	0.001
Distance to forest (df)	−0.152	0.042	−3.558	0.001
Distance to elephant reserves (der)	−0.466	0.041	−11.343	0.001
Distance to mines (dmn)	−0.244	0.040	−6.014	0.001
Distance to protected area (dpa)	0.298	0.040	7.346	0.001
Patch density (pd)	0.285	0.043	6.58	0.001
Distance to crop (dc)	0.072	0.05	1.446	0.15
Distance to built‐up (db)	1.138	0.063	17.879	0.001

### 3.3 HEC‐Village Level

**FIGURE 8:**
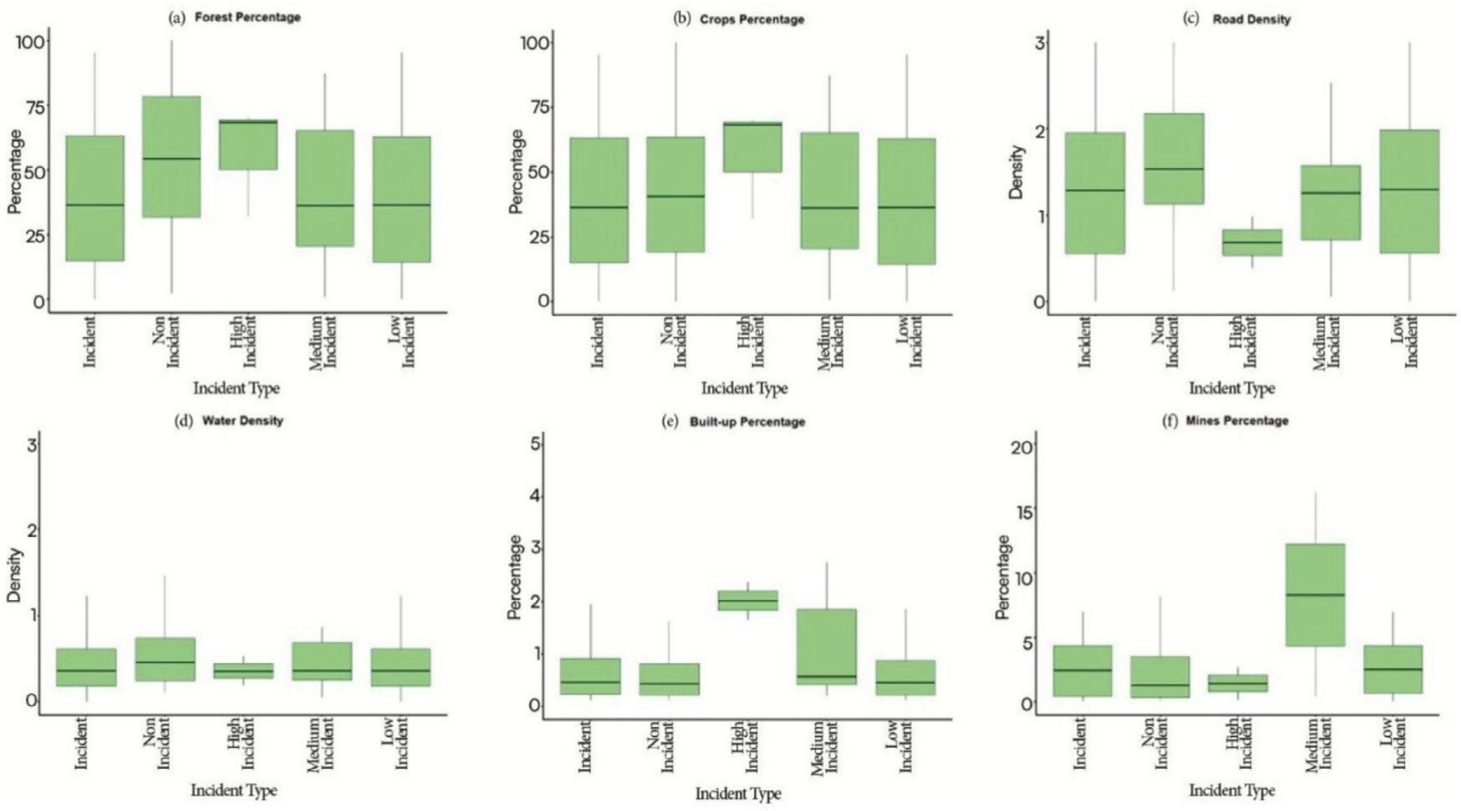
(a–f) Boxplot Illustrating the distribution of human fatalities and injuries across various incident categories, including incident villages, non‐incident villages, high incident villages, medium incident villages, low incident villages, providing insights into the variation in mortality levels across different regions of the state of Jharkhand, India from 2000 to 2023.

A detailed analysis of ecological and anthropogenic variables at the village level reveals important patterns across different conflict intensity categories. Non‐incident villages exhibit the highest median forest cover, while high‐conflict villages have relatively low forest cover compared to other conflict categories (Kruskal‐Wallis: *χ* ^2^ = 30.935, df = 4, *p* < 0.0001; Figure 8a). Post hoc Dunn test showed differences between incident and low‐incident villages (*p* adj. = 0.0001). High‐conflict villages show the highest cropland percentages, whereas medium and low‐conflict villages display broader variability in cropland cover (*χ* ^2^ = 11.403, df = 4, *p* = 0.022; Figure 8b). Road density is highest in high‐conflict villages, correlating with increased conflict intensity (*χ* ^2^ = 86.447, df = 4, *p* < 0.0001; Figure 8c). High‐conflict villages have the lowest water density, while medium‐conflict villages show variability, and low‐conflict villages demonstrate relatively stable water availability (*χ* ^2^ = 0.344, df = 4, *p* = 0.986; Figure 8d). Built‐up percentage is highest in high‐conflict villages and decreases with conflict intensity, with non‐incident villages having the lowest levels (*χ* ^2^ = 2.303, df = 4, *p* = 0.680; Figure 8e). Mining activities were lowest in high‐conflict villages, whereas medium‐conflict villages report the highest mining activity (*χ* ^2^ = 13.243, df = 2, *p* = 0.0013; Figure 8f).

## 4 Discussion

Human‐elephant conflict in Jharkhand has evolved from a sporadic issue to a more prevalent concern, reflecting global trends where human activities, particularly agricultural expansion and habitat fragmentation, are increasingly intersecting with elephant habitats, thereby escalating the frequency and intensity of conflicts (Sukumar 2006). The spatial distribution revealed a clear association between densely populated, agriculture‐dominated regions and conflict hotspots, which were concentrated in Ranchi, Khunti, and East Singhbhum. This is consistent with Khan et al. (2023), who also reported Ranchi and Khunti as key conflict hotspots driven by overlap between human activities and elephant habitats. The Dalma Wildlife Sanctuary and its surrounding areas, despite being a protected area, report a significant number of fatalities (Johnsingh and Williams 1999), emphasizing the challenges posed by such overlaps, which escalate the frequency of human‐elephant conflicts. Our spatial analysis highlights significant human encroachment in areas like Ranchi and Khunti, disrupting traditional elephant corridors and escalating HEC (Baskaran et al. 2013). These trends reflect broader patterns of HEC that have been documented in South Asia, particularly in regions where habitat fragmentation, agricultural expansion, and human settlement intersect with elephant habitats (Fernando et al. 2008; Baskaran et al. 2013; Pant et al. 2016). Similar trends have been reported in Assam, Odisha, and Sri Lanka, where human‐elephant conflict is exacerbated by encroachment into elephant corridors and protected areas (Fernando et al. 2005; Chartier et al. 2011; Guru and Das 2021). The east‐central region, including Jharkhand, has seen major dispersal of elephants from their former ranges (Pandey, Yadav, et al. 2024). Our results showed that fragmented landscapes intensify human–elephant encounters, echoing findings from Singhbhum ER, where numerous corridors contribute to elevated conflict. Despite conservation policies, elephant movement remains disrupted due to road and railway infrastructure. Approximately 1340 km of railway networks go through elephant habitats, leading to alterations in their movement patterns and increasing HEC, particularly in regions like Assam, Odisha, and Jharkhand (Pandey, Lakshminarayanan, et al. 2024). This is reflected in our findings, where high road density correlated with increased human fatalities, as disrupted corridors force elephants into closer contact with human settlements, intensifying conflicts.

The monsoon season in Jharkhand is a period having heightened HEC, which could be associated with the comparatively dense forests and reduced visibility during this time, making it more difficult for humans to spot elephants and leading to more frequent encounters. Together, these factors lead to a higher frequency of human–elephant encounters during the monsoon. Similar observations have been reported globally, where reduced visibility and seasonal habitat changes are known to elevate conflict (Karanth et al. 2013; Shaffer et al. 2019). Furthermore, our village‐level analysis of HEC across Jharkhand highlights the complex interactions between ecological and anthropogenic factors. Villages without conflict tend to have higher forest cover, supporting the idea that intact, undisturbed forests act as natural buffers, reducing human–elephant interactions. However, high‐conflict villages had relatively low forest cover, indicating that fragmentation forces elephants to traverse human‐ dominated spaces/landscapes, where they encounter people more frequently (Sukumar 2003). The presence of agricultural land, particularly in these villages, further exacerbates conflict as elephants are attracted to crops that are highly nutritious and energy rich compared to natural forage, particularly when fields occur adjacent to forest edges, aligning with findings on crop‐raiding behavior, confirming that agricultural activities attract elephants into human‐dominated landscape (Sukumar 2003; Montgomery et al. 2021). Road density also plays a significant role; high‐conflict villages show a strong positive correlation with road density, which fragments elephant habitats and disrupts traditional corridors, increasing the frequency of encounters (Sitati et al. 2003; Chaiyarat et al. 2022). Water availability also influenced conflict; high‐conflict villages had less water, supporting earlier findings that scarcity drives elephants into settlements (Naha et al. 2020; Gunawansa et al. 2023). In addition, percent built‐up areas, which are more prevalent in high‐conflict villages, further exacerbate the potential for conflict (Scrizzi et al. 2017; Fernando et al. 2023). These results highlight the multifaceted nature of HEC, shaped by both ecological conditions and human activities. Furthermore, our findings on the factors influencing human fatalities and injuries were largely consistent with the trends observed in the village‐level analysis. Proximity to water, roads, and forests all had negative associations with conflict, indicating that areas closer to water sources, roads, and fragmented habitats are more prone to HEC. This reflects the socioecological dynamics of Jharkhand's fragmented landscapes. Elephants, requiring substantial daily water intake, converge with people at limited water sources, particularly during the dry season, increasing the encounter risk. Roads cutting through elephant habitats fragment movement corridors and intensify human presence through settlements, transport, and agriculture, thereby increasing the likelihood of elephant–human encounters. Similarly, forest edges and fragmented patches serve as key contact zones where elephants moving between habitats or raiding crops encounter people engaged in farming or resource collection. Limited visibility in dense vegetation near water and forest fringes further heightens the risk of sudden, fatal encounters. These findings align with existing literature that highlights the role of water bodies in attracting elephants and roads in disrupting their movement (Dodd et al. 2024; Wilson et al. 2016; Shaffer et al. 2019). Our analysis indicates that conflict probability is higher in areas located close to mining activity and declines with increasing distance. This pattern can be explained by the extensive habitat loss and corridor disruption caused by open‐cast mining in Jharkhand and adjoining states like Odisha. Thousands of hectares of prime elephant habitat in Jharkhand and Odisha have been diverted for mining, leading to the destruction of traditional transit paths and forcing elephants into agricultural landscapes and villages, thereby intensifying human–elephant conflict (WPSI 2011). These findings highlight that mining landscapes act as conflict hotspots due to the combined effects of habitat fragmentation and dense human presence. In Jharkhand, elephants are not restricted to protected areas (PAs), resulting in a high incidence of conflicts even in regions far from PAs. However, as Elephant Reserves extend beyond PA boundaries, conflict incidence tends to decrease with increasing distance from these reserves. Conversely, conflict increases with distance from PAs, likely because most conflicts are concentrated along corridors and their surrounding areas, where elephant movement and human activities overlap significantly. Landscape fragmentation metrics, specifically patch density (number of forest patches per unit area, which captures the degree of fragmentation and landscape heterogeneity) and edge density (total length of forest–non‐forest boundaries per unit area), were also significant predictors, with higher fragmentation associated with increased conflict (Gubbi 2012; Karanth et al. 2012).

Overall, our results reinforce the need for spatially informed conflict mitigation strategies. Interventions should prioritize areas near water bodies, forest edges, roads, and mines to minimize conflict risk. Enhancing landscape connectivity, especially in fragmented forest patches, is crucial for ensuring safe passage for elephants while reducing human‐wildlife interactions. Additionally, regulating land‐use changes in conflict‐prone zones and incorporating local communities in management efforts will be vital for long‐term coexistence.

### 4.1 Limitations and Future Research Directions

Our study provides valuable insights into the spatial and ecological correlates of human–elephant conflict in Jharkhand. However, it is important to acknowledge certain limitations. The data were derived from compensation and incident records maintained by the Forest Department, which rarely document fine‐scale behavioral information about either elephants (e.g., group type, risk‐prone individuals, musth) or humans (e.g., crowd approaches, deterrent use). Consequently, our conclusions are necessarily structural and ecological rather than behavioral and mechanistic. This constrains our ability to explain how specific actions of humans or elephants translate into human fatalities, as emphasized by Mumby and Plotnik (2018), who argue for integrating cognition, individual variation, and behavioral dynamics into HEC mitigation.

Building on these limitations, future research should adopt an actor‐level perspective that complements structural analyses with direct behavioral observations. To strengthen conflict mitigation, studies need to move beyond structural drivers and systematically capture the behavioral dimensions of encounters, including how people respond during interactions, how elephants behave under different conditions, and the circumstances of visibility or crowding. Integrating such data into ecological and behavioral models would help explain why some encounters escalate into fatalities. Long‐term monitoring through camera traps, GPS‐collared elephants, and Rapid Response Team records can provide critical insights. Importantly, incorporating elephants' cognitive and sensory perspectives (Mumby and Plotnik 2018) will enable mitigation strategies that reduce escalation risk and ensure interventions are tailored, humane, and more effective than reliance on barriers alone. By explicitly integrating behavioral, cognitive, and ecological dimensions, future research can move toward a more comprehensive understanding of HEC and help design more targeted strategies to reduce human fatalities while ensuring elephant conservation.

## 5 Conclusion

Human–elephant conflict (HEC) in Jharkhand has intensified over the last two decades, shaped by habitat fragmentation, agricultural expansion, and increasing human activity in elephant ranges. Our analysis demonstrates that proximity to forests, water bodies, roads, mines, and elephant reserves, along with high levels of landscape fragmentation, significantly increases the risk of human fatalities and injuries. Hotspots were concentrated in Ranchi, Khunti, and East Singhbhum, while seasonal peaks during the monsoon suggest that visibility and ecological conditions further heighten risks.

These findings highlight the need for policy and management efforts to target priority areas for intervention. Priority should be given to high‐conflict villages such as Ramua, Chatambari, Gerapokhar, Gagi, Bhatin, and Koderma, where targeted strategies can have the greatest impact. Efforts should include restoring and maintaining habitat connectivity to reduce fragmentation (Rathnayake et al. 2022; Vasudev and Sukumar 2023), regulating land‐use change near critical conflict zones such as forest edges, croplands, and water sources (Pradhan et al. 2023; Nuwanjalee et al. 2023), and reducing road‐related disturbances through careful planning and mitigation structures such as solar fencing and trenches in Karnataka, India (Lingaraju and Saritha 2021; Anjum et al. 2023), and community‐maintained electric fences in Sri Lanka (TUI Wildlife Sri Lanka Project 2025; Community‐Based Seasonal Electric Fencing Programme 2024). In India, railway lines and highways have been repeatedly shown to fragment elephant corridors and cause direct mortalities, particularly in West Bengal, Assam, and southern India (Ahmed and Saikia 2022; Mitra 2017; Asian Nature Conservation Foundation and WWF 2019). Similar issues are documented in Sri Lanka, where elephants are frequently killed by trains and vehicle collisions on roads that bisect their ranges, making linear infrastructure one of the leading drivers of conflict (Mongabay India 2024; Harbingers Magazine 2025). Seasonal preparedness during the monsoon, including improved monitoring and early‐warning systems, will also be essential (MoEFCC 2019). During this period, heavy rainfall often reduces visibility and alters elephant movement patterns, increasing the likelihood of encounters with human settlements and agricultural areas. Community engagement must complement these ecological measures. Training and equipping local Rapid Response Teams, strengthening compensation and livelihood‐support schemes, and developing locally appropriate deterrents can help reduce direct risks to people. At the same time, strict regulation of mining and infrastructure expansion in sensitive zones is needed to address the structural drivers of conflict (Pradhan et al. 2023; Santiapillai and Jackson 1990; Wangdi and Stremme 2016; World Bank 2024).

In summary, our results show that HEC in Jharkhand is strongly linked to specific ecological and anthropogenic features, and therefore mitigation must be tailored to these drivers. By aligning interventions with spatial patterns of conflict risk, it is possible to reduce human fatalities while supporting long‐term elephant conservation and coexistence.

## Author Contributions

**Kalpana Roy:** data curation (equal), formal analysis (equal), validation (equal), visualization (equal), writing – original draft (equal), writing – review and editing (equal). **Ramesh Kumar Pandey:** conceptualization (equal), data curation (equal), formal analysis (equal), funding acquisition (equal), investigation (equal), project administration (equal), writing – review and editing (equal). **Athira N. Ganesan:** data curation (equal), formal analysis (equal), methodology (equal), visualization (equal), writing – original draft (equal), writing – review and editing (equal). **Ananya Dutta:** data curation (equal), visualization (equal), writing – review and editing (equal). **Dheeraj Mittal:** funding acquisition (equal), project administration (equal), validation (equal), writing – original draft (equal), writing – review and editing (equal). **Parag Nigam:** funding acquisition (equal), investigation (equal), project administration (equal), writing – review and editing (equal). **Anukul Nath:** data curation (equal), formal analysis (lead), methodology (equal), supervision (equal), validation (equal), visualization (equal), writing – original draft (equal), writing – review and editing (equal). **Bilal Habib:** conceptualization (lead), data curation (lead), formal analysis (lead), funding acquisition (lead), investigation (lead), methodology (lead), project administration (lead), supervision (lead), validation (lead), writing – original draft (lead), writing – review and editing (lead).

## Funding

This work was supported by Project Elephant Division, MoEFCC.

## Ethics Statement

The research is approved by the Animal and Social Ethics Committee of the Wildlife Institute of India.

## Conflicts of Interest

The authors declare no conflicts of interest.

## Supporting information


**Figure S1:** ece372679‐sup‐0001‐FigureS1.docx.

**Data S1:** ece372679‐sup‐0002‐DataS1.pdf.

## Data Availability

Data has been attached as a Supporting Information file (Data S1).
