# Supplementary figures and images for: Two Decades of Human–Elephant Conflict in Jharkhand: Spatial and Ecological Drivers of Human Fatalities
Source: Ecol Evol. 2025 Dec 18;15(12):e72679. doi: 10.1002/ece3.72679 (PMC12713083; doi:10.1002/ece3.72679)

**Supplementary Information**

**
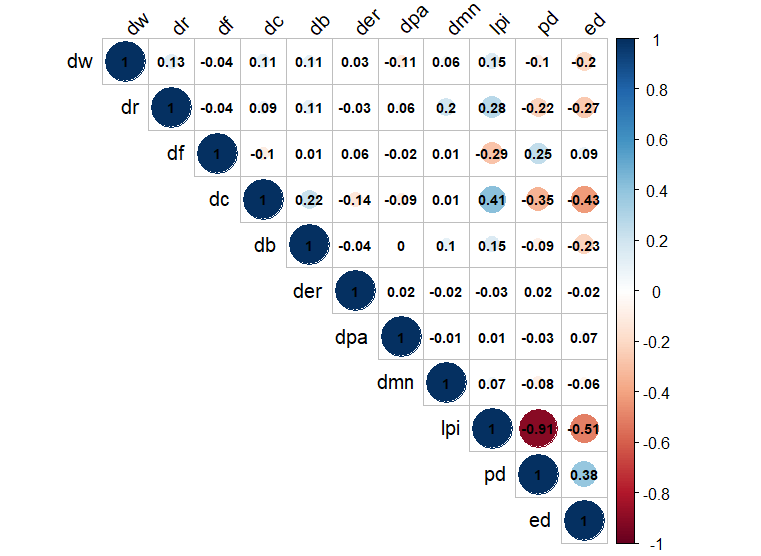
**

***SF1. Corelation matrix plot of the variables***

Supplement: Supplementary file 1 — Figure S1: ece372679‐sup‐0001‐FigureS1.docx. [file ECE3-15-e72679-s002.docx]
