# Supplementary material for: Two Decades of Human–Elephant Conflict in Jharkhand: Spatial and Ecological Drivers of Human Fatalities
Source: Ecol Evol. 2025 Dec 18;15(12):e72679. doi: 10.1002/ece3.72679 (PMC12713083; doi:10.1002/ece3.72679)
Supplement: Supplementary file 2 — Data S1: ece372679‐sup‐0002‐DataS1.pdf. [file ECE3-15-e72679-s001.pdf]

Villages in Jharkhand with recorded Human Mortality (2000-2023)

S.No.	Human Casu-alties Count	Village Name	Division	Admin-istrative level	Rural / Urban	House-hold	Popula-tion	Male%	Female%	Area (Ha)
1	218	Ranchi	Ranchi	Town	Urban	207636	1073427	52.06428	47.93572	15874.72
2	30	Titahia	Koderma	Village	Rural	79	498	50.80321	49.19679	80.41429
3	28	Gajgaon	Khunti	Village	Rural	199	1194	50.75377	49.24623	567.6787
4	28	Bramhaja-malpur	Sahibganj	Village	Rural	264	1378	51.88679	48.11321	601.6666
5	27	Bhu-chungdih	Ramgarh	Village	Rural	417	2309	50.49805	49.50195	605.9647
6	26	Sarbaha	Hazarib-agh	Village	Rural	479	2423	51.71275	48.28725	996.5635
7	26	Koinara	Ranchi	Village	Rural	254	1301	46.50269	53.49731	818.8382
8	25	Khokhro	Dalma Wildlife Sanctuary	Village	Rural	111	470	49.57447	50.42553	965.5186
9	24	Urikel	Khunti	Village	Rural	127	560	52.14286	47.85714	316.7189
10	23	Barinijkel	Khunti	Village	Rural	321	1670	50.71856	49.28144	1097.634
11	23	Kodarma	Koderma	Town	Urban	4337	24633	52.53522	47.46478	1505.811
12	22	Seraikella	Saraikela	Town	Urban	2975	14252	52.27337	47.72663	572.8355
13	22	Chandil CT	Saraikela	Town	Urban	1025	4839	51.66357	48.33643	110.9582
14	20	Dum	Dumka	Village	Rural	51	248	49.19355	50.80645	55.26032
15	19	Bisrampur	Saraikela	Village	Rural	91	488	49.18033	50.81967	309.0024
16	18	Khatanga	Khunti	Village	Rural	74	455	49.67033	50.32967	300.1453
17	18	Nagri	Koderma	Village	Rural	50	286	55.94406	44.05594	99.73123
18	18	Chamatu	Latehar	Village	Rural	340	1606	51.1208	48.8792	1254.863
19	17	Mahthadih	Koderma	Village	Rural	726	4084	51.42018	48.57982	127.3131
20	16	Tokisud	Dalma Wildlife Sanctuary	Village	Rural	183	997	50.45135	49.54865	1237.741
21	15	Chetar	Latehar	Village	Rural	143	810	50	50	474.1201
22	14	Meramhonar	West Singh-bhum	Village	Rural	503	2639	49.07162	50.92838	1063.11
23	14	Ichagarh	Saraikela	Village	Rural	358	1546	48.83571	51.16429	340.9982
24	13	Dimba	Ranchi	Village	Rural	276	1381	46.48805	53.51195	784.7551
25	12	Nagwan	Hazarib-agh	Village	Rural	626	3565	51.2763	48.7237	478.19
26	11	Meral	Khunti	Village	Rural	86	390	53.07692	46.92308	195.0083

27	11	Utrung	Khunti	Village	Rural	24	116	48.27586	51.72414	54.33768
28	11	Meghahatu-buru Forest Village	Saranda Forest Division	Town	Urban	1291	5992	52.62016	47.37984	228.8553
29	11	Gohla	East Sing-hbhum	Village	Rural	545	2545	50.13752	49.86248	658.4247
30	11	Lawa	Dalma Wildlife Sanctuary	Village	Rural	529	2487	49.33655	50.66345	378.6063
31	11	Pabira	Ranchi	Village	Rural	190	890	48.31461	51.68539	617.4809
32	10	Lota	Ranchi	Village	Rural	998	4760	50.77731	49.22269	443.7506
33	10	Lali	Ranchi	Village	Rural	663	3316	49.24608	50.75392	2440.937
34	10	Beti Benti	Ranchi	Village	Rural	265	1409	50.53229	49.46771	712.5114
35	10	Jarga	Ranchi	Village	Rural	344	1716	51.1655	48.8345	775.1774
36	9	Chargo	Giridih	Village	Rural	152	1017	49.36087	50.63913	315.8159
37	9	Dhangain	Palamu	Village	Rural	115	690	49.42029	50.57971	100.1152
38	9	Kolbonga	West Singh-bhum	Village	Rural	92	421	49.16865	50.83135	199.175
39	9	Hajam	Ranchi	Village	Rural	127	672	50.59524	49.40476	333.8552
40	9	Pandu	Ranchi	Village	Rural	131	705	47.94326	52.05674	457.0338
41	9	Danekera	Ranchi	Village	Rural	366	1764	48.12925	51.87075	1363.727
42	9	Nehalu-kapariya	Ranchi	Village	Rural	541	3344	50.77751	49.22249	1593.819
43	9	Gaubathan	Sahibganj	Village	Rural	0	0	0	0	65.83493
44	8	Gagi	Bokaro	Village	Rural	465	2667	51.93101	48.06899	89.28833
45	8	Kujukalan	Ramgarh	Village	Rural	73	463	51.83585	48.16415	284.5351
46	7	Gulhutu	Chatra	Village	Rural	28	139	48.20144	51.79856	153.4397
47	7	Katkamsanr	Hazarib-agh	Village	Rural	880	5132	50.83788	49.16212	1297.798
48	7	Marangburu	Khunti	Village	Rural	151	736	50.54348	49.45652	766.798
49	7	Itkithakur-goan	Ranchi	Village	Rural	2285	12174	50.44357	49.55643	571.9267
50	7	Kasari	Sahibganj	Village	Rural	36	155	55.48387	44.51613	115.6155
51	7	Panchkatiya	Sahibganj	Village	Rural	333	1563	51.82342	48.17658	271.0143
52	6	Topchanchi	Dhanbad	Town	Urban	1149	6082	51.11805	48.88195	345.1919
53	6	Telia Chak	Dumka	Village	Rural	113	561	52.94118	47.05882	26.91485
54	6	Turbunga	Gumla	Village	Rural	266	1346	49.10847	50.89153	731.2976
55	6	Hazaribag	Ranchi	Town	Urban	25794	142489	52.02647	47.97353	1596.269
56	6	Lipunga	West Singh-bhum	Village	Rural	138	749	48.33111	51.66889	1420.279
57	6	Lodhma	Ranchi	Village	Rural	224	1130	50.70796	49.29204	228.61
58	6	Katingkela	Ranchi	Village	Rural	193	1055	49.19431	50.80569	445.9878
59	6	Nimdih	Saraikela	Village	Rural	286	1349	52.48332	47.51668	102.2054
60	5	Simaria Khurd	Chatra	Village	Rural	190	1055	47.96209	52.03791	69.31765
61	5	Tundi	Dhanbad	Village	Rural	763	4221	48.96944	51.03056	199.0889
62	5	Gurdari	Gumla	Village	Rural	559	3432	54.31235	45.68765	2714.31
63	5	Ghatshila CT	East Sing-hbhum	Town	Urban	8893	40624	51.47696	48.52304	1359.188
64	5	Kontatola	Ranchi	Village	Rural	263	1213	51.4427	48.5573	596.4647
65	5	Ulatu	Ranchi	Village	Rural	1488	7812	50.47363	49.52637	4568.838
66	5	Latratu	Ranchi	Village	Rural	221	1175	50.7234	49.2766	938.4328
67	5	Bisa	Ranchi	Village	Rural	556	2976	50.70565	49.29435	1132.914
68	5	Bitaburu	Saraikela	Village	Rural	280	1338	51.34529	48.65471	416.7418
69	4	Kasmar	Bokaro	Village	Rural	587	2867	51.30799	48.69201	247.644
70	4	Pithakiyari	Dhanbad	Village	Rural	698	3506	52.53851	47.46149	37.12292

71	4	Tisri	Giridih	Village	Rural	680	3786	53.51294	46.48706	418.7352
72	4	Chappatoli	Gumla	Village	Rural	156	814	52.7027	47.2973	364.8031
73	4	Kunrwa	Hazarib-agh	Village	Rural	205	1208	49.83444	50.16556	544.7264
74	4	Khunti	Khunti	Town	Urban	7245	36390	51.01951	48.98049	2334.013
75	4	Chakulia	East Sing-hbhum	Town	Urban	3606	16306	51.22041	48.77959	1702.298
76	4	Kundaloka	East Sing-hbhum	Village	Rural	250	1265	49.72332	50.27668	736.1162
77	4	Paruliya	East Sing-hbhum	Village	Rural	1132	5286	49.65948	50.34052	1946.384
78	4	Duru	Ranchi	Village	Rural	119	606	51.9802	48.0198	347.8555
79	4	Kharsawan	Saraikela	Village	Rural	1192	5793	52.82237	47.17763	559.1485
80	4	Kandra	Saraikela	Town	Urban	1712	8157	51.96764	48.03236	279.3584
81	4	Parerkola	Sahibganj	Village	Rural	46	208	53.84615	46.15385	141.2531
82	3	Nawadih	Bokaro	Village	Rural	790	4362	50.82531	49.17469	209.8178
83	3	Tandwa	Chatra	Village	Rural	1126	6475	53.09653	46.90347	505.4252
84	3	Bakspura	Dhanbad	Village	Rural	425	2274	54.04573	45.95427	75.32242
85	3	Nipaniya	Godda	Village	Rural	586	2703	53.16315	46.83685	769.273
86	3	Cherra CT	Hazarib-agh	Town	Urban	953	5279	52.64255	47.35745	214.1345
87	3	Saraia	Hazarib-agh	Village	Rural	820	4855	50.7518	49.2482	1011.157
88	3	Sekhpura	Jamtara	Village	Rural	39	316	54.74684	45.25316	73.71273
89	3	Mihijam	Jamtara	Town	Urban	8139	40463	52.89029	47.10971	1055.077
90	3	Fatehpur	Jamtara	Village	Rural	841	4107	51.40005	48.59995	462.7602
91	3	Rengrahatu	West Singh-bhum	Village	Rural	345	1892	51.63848	48.36152	1444.487
92	3	Forest Block	East Sing-hbhum	Village	Rural	511	2665	48.93058	51.06942	4796.667
93	3	Mohanadi	East Sing-hbhum	Village	Rural	76	379	49.60422	50.39578	280.8072
94	3	Hehal	Ramgarh	Village	Rural	631	3808	51.65441	48.34559	479.9293
95	3	Patratu	Ramgarh	Town	Urban	6356	32899	52.60038	47.39962	2153.78
96	3	Bantara	Ramgarh	Village	Rural	300	1579	52.1216	47.8784	61.50309
97	3	Itihasa	Ranchi	Village	Rural	113	569	51.6696	48.3304	466.505
98	3	Lalganj	Ranchi	Village	Rural	382	1931	49.71517	50.28483	345.7354
99	3	Tati CT	Ranchi	Town	Urban	2544	12878	53.37009	46.62991	358.1733
100	3	Tumbaguttu	Ranchi	Village	Rural	562	3014	50.56403	49.43597	563.6805
101	3	Palandu	Ranchi	Village	Rural	211	1033	47.62827	52.37173	210.5759
102	3	Malti	Ranchi	Village	Rural	136	684	46.49123	53.50877	215.4549
103	3	Sakarpur	Ranchi	Village	Rural	181	1076	48.42007	51.57993	519.4541
104	3	Tilai	Ranchi	Village	Rural	72	408	48.52941	51.47059	288.6918
105	3	Biramkel	Ranchi	Village	Rural	116	663	49.4721	50.5279	364.2244
106	3	Dola	Ranchi	Village	Rural	76	426	51.40845	48.59155	428.0012
107	3	Sarugori	Ranchi	Village	Rural	202	880	51.59091	48.40909	463.7663
108	3	Sirka	Ranchi	Village	Rural	529	2550	51.05882	48.94118	554.4774
109	3	Barwadag	Ranchi	Village	Rural	250	1129	48.00709	51.99291	833.0239
110	3	Sitadih	Ranchi	Village	Rural	185	850	49.41176	50.58824	551.3599
111	3	Manipur	Saraikela	Village	Rural	66	300	52	48	200.307
112	3	Chati Go-bindpur	Dhanbad	Village	Rural	224	1355	49.7417	50.2583	25.68687
113	2	Chandan-kiari	Bokaro	Village	Rural	1831	9836	52.12485	47.87515	752.4467
114	2	Gumia CT	Bokaro	Town	Urban	9001	48141	52.17798	47.82202	3021.923
115	2	Bandh Dih CT	Bokaro	Town	Urban	2531	13192	53.03972	46.96028	1531.635

116	2	Partappur	Chatra	Village	Rural	717	3530	52.35127	47.64873	148.1146
117	2	Lawalong	Chatra	Village	Rural	67	347	53.60231	46.39769	387.1249
118	2	Majhipara	Chatra	Village	Rural	174	753	51.39442	48.60558	617.0536
119	2	Bariarchak	Chatra	Village	Rural	105	609	51.88834	48.11166	74.77489
120	2	Sikaripara	Dumka	Village	Rural	577	2646	50.90703	49.09297	182.0106
121	2	Ramgarh	Dumka	Village	Rural	191	961	49.21956	50.78044	104.9656
122	2	Jama	Dumka	Village	Rural	68	316	47.1519	52.8481	144.0977
123	2	Gopikandar	Dumka	Village	Rural	283	1293	50.58005	49.41995	146.9422
124	2	Jarmune	Giridih	Village	Rural	2616	15269	51.06425	48.93575	1143.615
125	2	Mohandih	Giridih	Village	Rural	296	1757	53.38645	46.61355	203.1836
126	2	Dhanwar Dakshin	Giridih	Town	Urban	1526	8777	51.62356	48.37644	201.9791
127	2	Harla	Giridih	Village	Rural	381	2332	51.84391	48.15609	164.1466
128	2	Chirki	Giridih	Village	Rural	425	2608	46.43405	53.56595	389.8284
129	2	Bara Boarijor	Godda	Village	Rural	452	2423	49.31903	50.68097	214.3052
130	2	Pareya Hat	Godda	Village	Rural	1294	6319	52.16015	47.83985	482.9034
131	2	Tajpur	Hazarib-agh	Village	Rural	782	4214	51.61367	48.38633	768.8329
132	2	Gopalpur	Jamtara	Village	Rural	357	1725	52.57971	47.42029	132.6035
133	2	Lappa	Ranchi	Village	Rural	277	1377	50.90777	49.09223	594.4689
134	2	Lumluma	Khunti	Village	Rural	87	452	50	50	195.6145
135	2	Barudih	Dalma Wildlife Sanctuary	Village	Rural	28	120	49.16667	50.83333	70.18098
136	2	Sisi	Latehar	Village	Rural	250	1345	48.77323	51.22677	486.4756
137	2	Babhandih	Palamu	Village	Rural	338	1755	52.53561	47.46439	466.9219
138	2	Nawa	Palamu	Village	Rural	248	1150	49.47826	50.52174	950.4031
139	2	Ladi	Palamu	Village	Rural	223	1232	53.8961	46.1039	136.1073
140	2	Sagalim	Palamu	Village	Rural	698	3577	49.98602	50.01398	471.1804
141	2	Kudaga Kalan	Palamu	Village	Rural	294	1434	52.51046	47.48954	217.1776
142	2	Baida	Palamu	Village	Rural	86	461	49.89154	50.10846	231.3245
143	2	Kamat Tola	Palamu	Village	Rural	42	204	46.56863	53.43137	15.85237
144	2	Chandarpur	Palamu	Village	Rural	130	827	51.39057	48.60943	143.402
145	2	Manatu	Palamu	Village	Rural	148	970	58.96907	41.03093	277.4978
146	2	Dumri	Palamu	Village	Rural	217	1271	53.81589	46.18411	968.1038
147	2	Rajhara	Palamu	Village	Rural	788	3958	50.90955	49.09045	1030.242
148	2	Teugri	Palamu	Village	Rural	70	332	53.31325	46.68675	60.18505
149	2	Hussainabad	Palamu	Town	Urban	4821	29241	52.24172	47.75828	1294.041
150	2	Baralota CT	Palamu	Town	Urban	2756	14880	53.40054	46.59946	704.9491
151	2	Bara	Palamu	Village	Rural	537	3342	51.94494	48.05506	213.2284
152	2	Adar	Palamu	Village	Rural	63	347	53.89049	46.10951	1079.641
153	2	Hisra	Palamu	Village	Rural	227	1044	48.27586	51.72414	899.9316
154	2	Haminpur	Palamu	Village	Rural	12	88	53.40909	46.59091	204.7385
155	2	Berma	Palamu	Village	Rural	444	2269	49.05245	50.94755	1298.37
156	2	Salatua	Palamu	Village	Rural	721	3583	50.96288	49.03712	1950.589
157	2	Belhara	Palamu	Village	Rural	217	1407	51.59915	48.40085	208.908
158	2	Bargua	West Singh-bhum	Village	Rural	23	99	44.44444	55.55556	9951.298
159	2	Kechabaipi	West Singh-bhum	Village	Rural	45	222	50.9009	49.0991	512.6183
160	2	Mohanpur	East Sing-hbhum	Village	Rural	428	1694	50.64935	49.35065	38.97547
161	2	Kantabani	East Sing-hbhum	Village	Rural	110	538	48.32714	51.67286	391.2531

162	2	Manikabera	East Sing-hbhum	Village	Rural	92	409	50.36675	49.63325	131.5326
163	2	Choira	East Sing-hbhum	Village	Rural	198	896	50.11161	49.88839	343.1496
164	2	Jambani	East Sing-hbhum	Village	Rural	111	496	55.04032	44.95968	70.61619
165	2	Dublabera	East Sing-hbhum	Village	Rural	128	654	49.38838	50.61162	560.1984
166	2	Galudi	East Sing-hbhum	Village	Rural	69	311	52.41158	47.58842	111.6411
167	2	Gobarghusi	Dalma Wildlife Sanctuary	Village	Rural	321	1482	50.53981	49.46019	1083.167
168	2	Dhusra	Dalma Wildlife Sanctuary	Village	Rural	213	993	50.25176	49.74824	432.8864
169	2	Khariyasai	East Sing-hbhum	Village	Rural	244	1146	48.51658	51.48342	273.9519
170	2	Gola	Ramgarh	Village	Rural	1527	8076	52.45171	47.54829	243.8634
171	2	Mandu CT	Ramgarh	Town	Urban	1926	10223	53.31116	46.68884	1742.537
172	2	Burhakhap	Ramgarh	Village	Rural	78	430	49.76744	50.23256	310.2396
173	2	Hariharpur	Ranchi	Village	Rural	238	1236	51.13269	48.86731	508.7891
174	2	Tunku	Ranchi	Village	Rural	262	1137	49.69217	50.30783	408.7146
175	2	Bantahajam	Ranchi	Village	Rural	1866	8677	50.66267	49.33733	2307.483
176	2	Chachgura	Ranchi	Village	Rural	259	1359	52.02355	47.97645	313.0669
177	2	Dharampur	Ranchi	Village	Rural	59	264	56.06061	43.93939	445.9045
178	2	Goradih	Ranchi	Village	Rural	574	2754	50.83515	49.16485	823.3277
179	2	Baridih	Ranchi	Village	Rural	230	1304	49.76994	50.23006	251.9975
180	2	Basantpur	Ranchi	Village	Rural	361	1716	48.71795	51.28205	237.8993
181	2	Purnadih	Ranchi	Village	Rural	89	464	52.37069	47.62931	376.4056
182	2	Kuchu	Ranchi	Village	Rural	459	2371	51.83467	48.16533	474.8332
183	2	Bhusur	Ranchi	Village	Rural	148	745	50.4698	49.5302	316.7909
184	2	Damari	Ranchi	Village	Rural	324	1436	52.08914	47.91086	91.96449
185	2	Jaratoli	Ranchi	Village	Rural	47	250	52.8	47.2	180.9261
186	2	Kota	Ranchi	Village	Rural	207	1244	50.5627	49.4373	203.1911
187	2	Piska	Ranchi	Village	Rural	188	954	48.11321	51.88679	305.008
188	2	Karum	Ranchi	Village	Rural	113	584	51.5411	48.4589	144.758
189	2	Malgo	Ranchi	Village	Rural	433	2248	49.33274	50.66726	874.8487
190	2	Jataloya	Ranchi	Village	Rural	184	958	48.32985	51.67015	350.419
191	2	Sarangloya	Ranchi	Village	Rural	123	651	48.69432	51.30568	385.4161
192	2	Pithauriya	Ranchi	Village	Rural	1243	6550	52.10687	47.89313	451.0853
193	2	Kuli	Ranchi	Village	Rural	571	3208	49.15835	50.84165	1033.237
194	2	Bhandra	Ranchi	Village	Rural	232	1519	51.4154	48.5846	343.4898
195	2	Chatwal	Ranchi	Village	Rural	399	2447	50.91949	49.08051	214.1696
196	2	Sero	Ranchi	Village	Rural	422	2278	50.26339	49.73661	760.5367
197	2	Tuko	Ranchi	Village	Rural	372	2270	51.4978	48.5022	395.592
198	2	Mahru	Ranchi	Village	Rural	117	687	51.96507	48.03493	253.1499
199	2	Jahanabaj	Ranchi	Village	Rural	269	1346	50.14859	49.85141	491.9362
200	2	Koynardih	Ranchi	Village	Rural	183	826	48.30508	51.69492	793.5235
201	2	Badri	Ranchi	Village	Rural	288	1438	51.32128	48.67872	897.3612
202	2	Palna	Saraikela	Village	Rural	171	843	49.58482	50.41518	397.6321
203	2	Chowka	Saraikela	Village	Rural	366	1492	52.27882	47.72118	132.4728
204	2	Raghunath-pur	Saraikela	Village	Rural	759	3351	50.88033	49.11967	381.3297
205	2	Sirkadih	Saraikela	Village	Rural	182	802	49.62594	50.37406	101.349
206	2	Rajnagar	Saraikela	Village	Rural	390	1806	49.83389	50.16611	207.4843
207	2	Raipur	Saraikela	Village	Rural	131	668	50.8982	49.1018	146.618

208	2	Komdih	West Singh-bhum	Village	Rural	89	660	45.60606	54.39394	141.3098
209	2	Kandarbera	Saraikela	Village	Rural	266	1159	50.38827	49.61173	665.6411
210	2	Rudih	Saraikela	Village	Rural	466	1957	51.25192	48.74808	687.1544
211	2	Nawapara	Sahibganj	Village	Rural	167	833	49.45978	50.54022	309.1973
212	1	Chandrapu-ra CT	Bokaro	Town	Urban	5520	27425	52.60893	47.39107	710.0745
213	1	Chas	Bokaro	Town	Urban	25540	141640	52.7584	47.2416	1890.515
214	1	Uda	Bokaro	Village	Rural	70	352	50.85227	49.14773	568.2545
215	1	Kalyanpur	Bokaro	Village	Rural	456	2105	50.30879	49.69121	682.7964
216	1	Jhirki	Ranchi	Village	Rural	171	821	52.00974	47.99026	128.4209
217	1	Khutikewal Khurd	Chatra	Village	Rural	452	2372	50.92749	49.07251	94.66339
218	1	Gidhaur	Chatra	Village	Rural	1021	5520	51.86594	48.13406	1451.751
219	1	Sagdaha	Ranchi	Village	Rural	181	1026	53.9961	46.0039	157.859
220	1	Dhanbad	Dhanbad	Town	Urban	220783	1162472	52.88059	47.11941	21639.64
221	1	Sihulibana	Dalma Wildlife Sanctuary	Village	Rural	114	535	53.08411	46.91589	250.2328
222	1	Dongo	Giridih	Village	Rural	43	392	49.4898	50.5102	196.4215
223	1	Asgando	Giridih	Village	Rural	0	0	0	0	215.1235
224	1	Karodih	Giridih	Village	Rural	220	1632	51.59314	48.40686	511.8262
225	1	Sundar Pahari	Godda	Village	Rural	196	888	41.21622	58.78378	436.4776
226	1	Dumberi Kajri	Godda	Village	Rural	12	39	58.97436	41.02564	60.6862
227	1	Basantpur	Godda	Village	Rural	129	676	49.55621	50.44379	16.44154
228	1	Chamru Kaprigangti	Godda	Village	Rural	64	400	52.25	47.75	52.77691
229	1	Duarichak	Godda	Village	Rural	238	1150	54.08696	45.91304	36.25238
230	1	Banka Ghat	Godda	Village	Rural	631	3092	51.94049	48.05951	555.8086
231	1	Godda	Godda	Town	Urban	8969	48480	53.02599	46.97401	793.1708
232	1	Lunga	Gumla	Village	Rural	89	401	46.63342	53.36658	316.6319
233	1	Nawagarh	Gumla	Village	Rural	1231	6529	48.30755	51.69245	2968.596
234	1	Tigawal	Gumla	Village	Rural	207	1209	49.54508	50.45492	887.9088
235	1	Korekera	Gumla	Village	Rural	186	863	46.34994	53.65006	488.5699
236	1	Bhagidera	Ranchi	Village	Rural	342	1644	50.36496	49.63504	121.1934
237	1	Basiya	Gumla	Village	Rural	821	3985	49.66123	50.33877	983.1025
238	1	Palkot	Gumla	Village	Rural	1753	8945	49.33482	50.66518	2728.008
239	1	Beyar	Gumla	Village	Rural	112	662	50	50	634.9645
240	1	Ruki	Gumla	Village	Rural	233	1268	50.07886	49.92114	767.0739
241	1	Besna	Gumla	Village	Rural	80	504	53.37302	46.62698	572.4143
242	1	Rajadera	Gumla	Village	Rural	52	344	48.83721	51.16279	785.3384
243	1	Harsari	Gumla	Village	Rural	176	901	50.83241	49.16759	476.6018
244	1	Barhi CT	Hazarib-agh	Town	Urban	2011	11867	51.46204	48.53796	379.9937
245	1	Barkagaon	Hazarib-agh	Village	Rural	2063	11689	51.52708	48.47292	341.4436
246	1	Barahkatha	Hazarib-agh	Village	Rural	1445	8364	52.05643	47.94357	370.5233
247	1	Masipirhi	Hazarib-agh	Village	Rural	281	1799	51.13952	48.86048	358.1209
248	1	Churchu	Hazarib-agh	Village	Rural	246	1279	52.77561	47.22439	335.8683
249	1	Dari CT	Hazarib-agh	Town	Urban	1219	6405	51.25683	48.74317	1098.509
250	1	Parasi	Hazarib-agh	Village	Rural	591	3211	52.94301	47.05699	317.2811

251	1	Keredari	Hazarib-agh	Village	Rural	716	3693	51.77363	48.22637	907.6293
252	1	Padma	Hazarib-agh	Village	Rural	1407	7896	54.78723	45.21277	898.3637
253	1	Aintha	Hazarib-agh	Village	Rural	42	260	54.61538	45.38462	100.3765
254	1	Daldala	Jamtara	Village	Rural	101	494	51.21457	48.78543	103.0872
255	1	Muraam	Jamtara	Village	Rural	117	689	54.86212	45.13788	253.8185
256	1	Mahulbana	Jamtara	Village	Rural	306	1464	51.02459	48.97541	381.6658
257	1	Teliadi	Jamtara	Village	Rural	164	765	51.24183	48.75817	84.35936
258	1	Karma Tanr CT	Jamtara	Town	Urban	1082	5868	51.36333	48.63667	294.6071
259	1	Rampur Bhitra	Jamtara	Village	Rural	78	488	51.02459	48.97541	179.2819
260	1	Raghunath-pur	Jamtara	Village	Rural	5	32	50	50	20.85273
261	1	Jorbhitha	Jamtara	Village	Rural	109	529	47.82609	52.17391	271.5377
262	1	Kushmap-ahari	Jamtara	Village	Rural	92	488	51.63934	48.36066	156.9332
263	1	Posai	Jamtara	Village	Rural	209	1060	52.92453	47.07547	153.8287
264	1	Jamtara	Jamtara	Town	Urban	5743	29415	52.25905	47.74095	1377.067
265	1	Kumhardih	Khunti	Village	Rural	78	569	51.84534	48.15466	437.6519
266	1	Jilingkel	Khunti	Village	Rural	210	1128	50.26596	49.73404	776.9331
267	1	Kudapurti	Khunti	Village	Rural	391	2027	50.66601	49.33399	1804.92
268	1	Hunth	Khunti	Village	Rural	225	1242	49.75845	50.24155	557.1155
269	1	Chuklu	Khunti	Village	Rural	267	1392	50.71839	49.28161	1322.863
270	1	Raytorang	Khunti	Village	Rural	77	391	47.82609	52.17391	411.2485
271	1	Ronhi	Ranchi	Village	Rural	117	584	48.9726	51.0274	231.37
272	1	Galiondar	Khunti	Village	Rural	59	350	49.71429	50.28571	287.1411
273	1	Dumargari	Ranchi	Village	Rural	261	1235	48.34008	51.65992	950.2836
274	1	Kadal	Gumla	Village	Rural	78	455	47.47253	52.52747	501.8252
275	1	Taski	Ranchi	Village	Rural	125	683	50.51245	49.48755	210.8015
276	1	Timra	Ranchi	Village	Rural	114	741	47.36842	52.63158	190.1979
277	1	Jojodag	Ranchi	Village	Rural	149	622	49.03537	50.96463	202.2641
278	1	Suti	Ranchi	Village	Rural	66	323	48.60681	51.39319	117.1047
279	1	Korakel	Khunti	Village	Rural	519	2489	49.57814	50.42186	423.2207
280	1	Hethgowa	Khunti	Village	Rural	292	1565	50.35144	49.64856	645.7577
281	1	Sirka	Khunti	Village	Rural	183	913	50.60241	49.39759	842.4586
282	1	Selda	Khunti	Village	Rural	90	509	51.8664	48.1336	251.7669
283	1	Bundu Mamail	Khunti	Village	Rural	97	572	53.14685	46.85315	717.7468
284	1	Guruburu	Khunti	Village	Rural	46	218	51.37615	48.62385	101.9987
285	1	Dembukel	Khunti	Village	Rural	101	520	48.84615	51.15385	453.5486
286	1	Kelo	Khunti	Village	Rural	89	416	47.83654	52.16346	207.2752
287	1	Okra	Ranchi	Village	Rural	412	2184	49.03846	50.96154	1030.026
288	1	Dumangdiri	Khunti	Village	Rural	66	398	49.49749	50.50251	191.8394
289	1	Anigara	Khunti	Village	Rural	394	2071	49.44471	50.55529	763.0735
290	1	Sandasom	Khunti	Village	Rural	160	791	52.33881	47.66119	426.2283
291	1	Khirikala	Koderma	Village	Rural	23	108	58.33333	41.66667	154.9877
292	1	Mail	Latehar	Village	Rural	487	2885	51.43847	48.56153	1085.08
293	1	Hundru	Latehar	Village	Rural	213	1149	50.82681	49.17319	332.3284
294	1	Baritu Jagir	Latehar	Village	Rural	257	1261	49.64314	50.35686	308.9705
295	1	Betla	Palamu	Village	Rural	432	2193	52.30278	47.69722	618.2215
296	1	Kharta	Ranchi	Village	Rural	284	1578	49.42966	50.57034	614.8854
297	1	Sitagarh	Pakur	Village	Rural	82	430	49.30233	50.69767	126.6353
298	1	Kasila	Pakur	Village	Rural	383	1919	48.82751	51.17249	302.6543

299	1	Anupdanga	Pakur	Village	Rural	147	766	50.78329	49.21671	279.6251
300	1	Jirli	Pakur	Village	Rural	229	1057	48.06055	51.93945	257.8852
301	1	Dasgora	Pakur	Village	Rural	95	397	42.06549	57.93451	349.4873
302	1	Champa	Pakur	Village	Rural	24	102	50.98039	49.01961	129.0564
303	1	Karmatanr	Pakur	Village	Rural	157	716	51.11732	48.88268	315.2824
304	1	Nerpahari	Pakur	Village	Rural	2	12	50	50	23.93392
305	1	Telopara	Pakur	Village	Rural	102	494	50.80972	49.19028	173.3952
306	1	Kidirpur	Pakur	Village	Rural	187	875	51.65714	48.34286	91.55354
307	1	Talpahari	Pakur	Village	Rural	173	784	50.12755	49.87245	252.6651
308	1	Gorpara	Pakur	Village	Rural	115	559	50.9839	49.0161	421.6843
309	1	Kundamatia	Pakur	Village	Rural	95	413	52.7845	47.2155	282.2377
310	1	Ghaghri	West Singh-bhum	Village	Rural	251	1263	47.26841	52.73159	477.5853
311	1	Kebedkara	West Singh-bhum	Village	Rural	193	983	52.18718	47.81282	270.9827
312	1	Sangajata	West Singh-bhum	Village	Rural	82	410	50	50	486.8949
313	1	Jojohatu	West Singh-bhum	Village	Rural	94	437	51.25858	48.74142	259.264
314	1	Thaikobad	West Singh-bhum	Village	Rural	76	320	55.625	44.375	221.5167
315	1	Kudripa	West Singh-bhum	Village	Rural	47	266	47.36842	52.63158	5106.536
316	1	Thakura	West Singh-bhum	Village	Rural	140	694	49.71182	50.28818	1242.543
317	1	Patamda	East Sing-hbhum	Village	Rural	511	2558	52.15012	47.84988	385.1854
318	1	Boram	East Sing-hbhum	Village	Rural	664	3314	53.10803	46.89197	425.2204
319	1	Laylam	Dalma Wildlife Sanctuary	Village	Rural	330	1673	51.46444	48.53556	1086.947
320	1	Muturkham	East Sing-hbhum	Village	Rural	142	650	49.23077	50.76923	352.0246
321	1	Swargach-hinra	East Sing-hbhum	Village	Rural	89	389	50.64267	49.35733	144.3419
322	1	Mahisadhara	East Sing-hbhum	Village	Rural	110	524	50.38168	49.61832	438.5321
323	1	Behra	East Sing-hbhum	Village	Rural	178	768	49.47917	50.52083	212.8206
324	1	Katashol	East Sing-hbhum	Village	Rural	164	865	54.21965	45.78035	456.8466
325	1	Bankishol	East Sing-hbhum	Village	Rural	428	2001	49.42529	50.57471	621.388
326	1	Jangle Block	East Sing-hbhum	Village	Rural	109	622	51.76849	48.23151	1851.488
327	1	Kalimati	East Sing-hbhum	Village	Rural	82	434	47.92627	52.07373	342.0428
328	1	Manpita	Dalma Wildlife Sanctuary	Village	Rural	434	2207	50.11328	49.88672	325.175
329	1	Jadugora CT	East Sing-hbhum	Town	Urban	3755	18563	51.34407	48.65593	409.7298
330	1	Sohada	East Sing-hbhum	Village	Rural	585	2754	51.08932	48.91068	368.9916
331	1	Musabani CT	East Sing-hbhum	Town	Urban	6650	31035	51.75769	48.24231	1344.325

332	1	Bikrampur Alias Main-jhariya	East Sing-hbhum	Village	Rural	282	1381	48.51557	51.48443	764.7763
333	1	Kakdaha	East Sing-hbhum	Village	Rural	254	1233	50.36496	49.63504	1029.891
334	1	Sindurgauri	East Sing-hbhum	Village	Rural	41	190	50	50	105.796
335	1	Tilaitanr	East Sing-hbhum	Village	Rural	248	1203	48.5453	51.4547	68.62268
336	1	Dabanki	East Sing-hbhum	Village	Rural	219	1071	51.07376	48.92624	228.6495
337	1	Saharjuri	East Sing-hbhum	Village	Rural	65	321	48.59813	51.40187	556.1022
338	1	Harina	East Sing-hbhum	Village	Rural	294	1359	51.21413	48.78587	419.1471
339	1	Telaidi	East Sing-hbhum	Village	Rural	52	236	47.88136	52.11864	188.2694
340	1	Roteda	East Sing-hbhum	Village	Rural	38	169	44.97041	55.02959	62.54537
341	1	Ghaghra	Ramgarh	Village	Rural	111	629	49.92051	50.07949	446.2594
342	1	Ramgarh Nagar Pari-shad	Ramgarh	Town	Urban	16592	88781	54.18952	45.81048	2952.905
343	1	Gorha	Ranchi	Village	Rural	139	623	53.29053	46.70947	472.0553
344	1	Dowaru	Ranchi	Village	Rural	684	2989	50.68585	49.31415	1984.939
345	1	Lawadag	Ranchi	Village	Rural	207	966	51.34576	48.65424	148.0887
346	1	Chatambari	Ranchi	Village	Rural	154	706	50	50	493.3335
347	1	Patratu	Ranchi	Village	Rural	124	678	47.34513	52.65487	188.8936
348	1	Birgaon	Ranchi	Village	Rural	863	3565	50.57504	49.42496	1166.985
349	1	Jaluhutang	Ranchi	Village	Rural	155	706	48.86686	51.13314	498.5604
350	1	Jumla	Ranchi	Village	Rural	109	546	49.6337	50.3663	427.7834
351	1	Bhuli	Ranchi	Village	Rural	73	367	49.04632	50.95368	99.51224
352	1	Lagam	Ranchi	Village	Rural	356	1837	50.2994	49.7006	157.3246
353	1	Silli	Ranchi	Village	Rural	1072	5222	51.39793	48.60207	236.8513
354	1	Tutki	Ranchi	Village	Rural	434	2048	50.24414	49.75586	364.0558
355	1	Madni	Ranchi	Village	Rural	154	742	49.0566	50.9434	385.7595
356	1	Ratu CT	Ranchi	Town	Urban	4434	22379	51.4813	48.5187	929.7835
357	1	Tilta	Ranchi	Village	Rural	350	1839	50.08157	49.91843	260.0563
358	1	Nauasoso	Ranchi	Village	Rural	215	1116	50.98566	49.01434	155.2945
359	1	Bansiya	Ranchi	Village	Rural	395	1817	50.96313	49.03687	736.0887
360	1	Ladhup	Ranchi	Village	Rural	339	1445	50.38062	49.61938	643.6979
361	1	Guchidih	Ranchi	Village	Rural	189	782	50	50	179.8443
362	1	Dunde	Ranchi	Village	Rural	71	375	52.53333	47.46667	98.3014
363	1	Mahilong	Ranchi	Village	Rural	917	4371	50.90368	49.09632	846.5363
364	1	Arma	Ranchi	Village	Rural	0	0	0	0	38.74858
365	1	Tetri	Ranchi	Village	Rural	299	1473	51.79905	48.20095	467.2017
366	1	Baredih	Ranchi	Village	Rural	271	1262	52.6149	47.3851	282.2074
367	1	Sukurhuttu	Ranchi	Village	Rural	206	1112	48.56115	51.43885	143.6137
368	1	Jaipur	Ranchi	Village	Rural	177	1008	50	50	149.3348
369	1	Saher	Ranchi	Village	Rural	587	3084	51.32944	48.67056	559.9153
370	1	Sugda	Ranchi	Village	Rural	123	714	50.28011	49.71989	134.0589
371	1	Rege	Ranchi	Village	Rural	115	624	50.48077	49.51923	108.764
372	1	Nagra	Ranchi	Village	Rural	943	5929	49.67111	50.32889	1068.706
373	1	Kanijari	Ranchi	Village	Rural	184	1089	47.56657	52.43343	310.7493
374	1	Taberkalan	Ranchi	Village	Rural	54	303	50.16502	49.83498	238.6713
375	1	Balandu	Ranchi	Village	Rural	189	1008	49.60317	50.39683	190.8343
376	1	Sarsa	Ranchi	Village	Rural	205	1163	49.26913	50.73087	641.7799

377	1	Pola	Ranchi	Village	Rural	126	736	48.77717	51.22283	482.5043
378	1	Tetra	Ranchi	Village	Rural	113	618	51.77994	48.22006	166.1588
379	1	Koisara	Ranchi	Village	Rural	193	831	48.25511	51.74489	353.3712
380	1	Fatehpur	Ranchi	Village	Rural	210	1126	49.02309	50.97691	300.3878
381	1	Lapung	Ranchi	Village	Rural	192	903	50.94131	49.05869	258.5996
382	1	Chalha	Ranchi	Village	Rural	42	230	50.86957	49.13043	107.1567
383	1	Churi CT	Ranchi	Town	Urban	4972	24876	52.23911	47.76089	1670.16
384	1	Khelari CT	Ranchi	Town	Urban	3800	20010	51.72914	48.27086	1744.283
385	1	Pusu	Ranchi	Village	Rural	172	888	49.66216	50.33784	791.9364
386	1	Jidu	Ranchi	Village	Rural	92	508	39.96063	60.03937	187.7839
387	1	Marwa	Ranchi	Village	Rural	268	1274	49.68603	50.31397	777.9051
388	1	Jaipur	Ranchi	Village	Rural	471	2560	51.28906	48.71094	160.6899
389	1	Sukurhuttu	Ranchi	Village	Rural	2118	11862	52.91688	47.08312	1148.601
390	1	Soso	Ranchi	Village	Rural	200	1035	52.07729	47.92271	230.026
391	1	Chinaro	Ranchi	Village	Rural	84	523	48.94837	51.05163	194.6014
392	1	Puriya	Ranchi	Village	Rural	116	733	51.29604	48.70396	643.1801
393	1	Ara	Ranchi	Village	Rural	114	510	52.15686	47.84314	480.8171
394	1	Kamta	Ranchi	Village	Rural	483	2401	49.77093	50.22907	460.1318
395	1	Harra	Ranchi	Village	Rural	172	899	50.16685	49.83315	277.5945
396	1	Madai	Ranchi	Village	Rural	114	731	50.75239	49.24761	134.7577
397	1	Umedanda	Ranchi	Village	Rural	675	3644	51.75631	48.24369	1242.659
398	1	Gurgain	Ranchi	Village	Rural	558	3008	50.26596	49.73404	625.7144
399	1	Kotari	Ranchi	Village	Rural	165	843	51.24555	48.75445	470.8374
400	1	Nawadih	Ranchi	Village	Rural	49	225	52.44444	47.55556	77.86024
401	1	Murto	Ranchi	Village	Rural	349	2063	51.04217	48.95783	462.4116
402	1	Karanji	Ranchi	Village	Rural	538	3119	51.07406	48.92594	413.6117
403	1	Bero	Ranchi	Village	Rural	1557	7193	48.89476	51.10524	671.2505
404	1	Singari	Ranchi	Village	Rural	386	2025	49.58025	50.41975	633.3831
405	1	Sursu	Ranchi	Village	Rural	414	2026	43.97828	56.02172	1400.86
406	1	Malghong-hsa	Ranchi	Village	Rural	369	1814	50.60639	49.39361	946.4913
407	1	Hesatu	Ranchi	Village	Rural	620	3161	52.76811	47.23189	569.5298
408	1	Soso	Ranchi	Village	Rural	210	1124	50.97865	49.02135	333.32
409	1	Jaradih	Ranchi	Village	Rural	186	955	51.72775	48.27225	361.0819
410	1	Dahua	Ranchi	Village	Rural	112	556	50.89928	49.10072	141.1334
411	1	Kashidih	Ranchi	Village	Rural	181	822	51.94647	48.05353	566.9034
412	1	Mungadih	Ranchi	Village	Rural	63	327	49.84709	50.15291	185.8356
413	1	Jonha	Ranchi	Village	Rural	414	1982	47.32593	52.67407	335.2686
414	1	Heslabera	Ranchi	Village	Rural	264	1430	50.27972	49.72028	652.3927
415	1	Kachojara	Ranchi	Village	Rural	18	86	58.13953	41.86047	211.3182
416	1	Sindri	Saraikela	Village	Rural	170	769	55.6567	44.3433	176.2343
417	1	Jhimiri	Dalma Wildlife Sanctuary	Village	Rural	731	3381	52.26264	47.73736	532.2888
418	1	Bandhdih	Dalma Wildlife Sanctuary	Village	Rural	186	851	50.88132	49.11868	946.2175
419	1	Edaldih	Saraikela	Village	Rural	92	341	48.97361	51.02639	100.0266
420	1	Kuchai	Saraikela	Village	Rural	247	1315	56.12167	43.87833	379.3515
421	1	Raijama	Saraikela	Village	Rural	161	752	51.59574	48.40426	1058.782
422	1	Kashidih	Saraikela	Village	Rural	53	284	56.69014	43.30986	295.6404
423	1	Raidih	Saraikela	Village	Rural	133	593	52.27656	47.72344	279.3857
424	1	Sauntari	Saraikela	Village	Rural	158	777	51.09395	48.90605	165.6433
425	1	Khelarisai	Saraikela	Village	Rural	136	665	52.18045	47.81955	184.9167
426	1	Bandu	Saraikela	Village	Rural	390	1773	51.77665	48.22335	374.8331

427	1	Jamdih	Saraikela	Village	Rural	134	824	47.93689	52.06311	304.2961
428	1	Mahuldiha	Saraikela	Village	Rural	49	230	47.3913	52.6087	85.68882
429	1	Ramgarh	Saraikela	Village	Rural	153	752	52.26064	47.73936	690.4422
430	1	Kopali CT	Saraikela	Town	Urban	7633	43256	51.63908	48.36092	645.1824
431	1	Dobo	Saraikela	Village	Rural	466	2235	49.61969	50.38031	566.6225
432	1	Pusisili	Saraikela	Village	Rural	278	1262	50.31696	49.68304	478.7727
433	1	Saharbera	Saraikela	Village	Rural	304	1492	52.27882	47.72118	531.385
434	1	Balrampur	Saraikela	Village	Rural	826	3893	53.0953	46.9047	176.6148
435	1	Adityapur	Saraikela	Town	Urban	37206	174355	52.5732	47.4268	4877.479
436	1	Sakarpada	Ranchi	Village	Rural	249	1381	50.76032	49.23968	185.8047
437	1	Hurhuru	Hazarib-agh	Village	Rural	357	1929	49.45568	50.54432	355.5526
438	1	Sirsi No I	Hazarib-agh	Village	Rural	291	1621	50.64775	49.35225	92.32478
439	1	Buti	Ranchi	Village	Rural	33	150	51.33333	48.66667	140.4353
440	1	Harwadih	Ranchi	Village	Rural	195	836	49.16268	50.83732	233.3049
441	1	Masna Miapur	Sahibganj	Village	Rural	0	0	0	0	212.9064
442	1	Komodari	Sahibganj	Village	Rural	40	191	51.3089	48.6911	198.7904
443	1	Bistupur	Sahibganj	Village	Rural	189	1006	49.50298	50.49702	325.728
444	1	Banjhikend	Sahibganj	Village	Rural	116	487	47.22793	52.77207	77.34815
445	1	Jetkekumar-jori	Sahibganj	Village	Rural	195	914	50.76586	49.23414	285.2409
446	1	Sitalpur	Sahibganj	Village	Rural	70	332	51.50602	48.49398	213.4793
447	1	Pokria	Sahibganj	Village	Rural	22	96	55.20833	44.79167	150.1582
448	1	Tilaki	Sahibganj	Village	Rural	123	624	53.20513	46.79487	206.2552
449	1	Sabda Chhota Gargram	Sahibganj	Village	Rural	197	826	53.1477	46.8523	129.0961
450	1	Satrampur	Sahibganj	Village	Rural	0	0	0	0	99.67424
451	1	Risor	Sahibganj	Village	Rural	800	4007	50.08735	49.91265	519.9643
452	1	Mirjapur Khurd	Sahibganj	Village	Rural	0	0	0	0	55.45004
453	1	Maheshghati	Sahibganj	Village	Rural	477	2219	50.15773	49.84227	192.6313
